# Economic geography of innovation: The effect of gender-related aspects of co-inventor networks on country and regional innovation

**DOI:** 10.1371/journal.pone.0288843

**Published:** 2023-07-27

**Authors:** Leila Tahmooresnejad, Ekaterina Turkina

**Affiliations:** HEC Montréal, Montréal, Quebec, Canada; Unviersity of Burgundy, FRANCE

## Abstract

This paper focuses on the analysis of the effects of inventor networks on country and regional innovation. We use data from an OECD inventor database that spans more than forty years to build collaboration networks in which the network nodes are countries and regions, and linkages are patents produced by inventors from different regions and countries. We first investigate the network that includes all inventors and then analyze the network focusing on women inventors. We argue that both country and regional-level network centrality positively affect country and regional innovation (with stronger effects at the country level), and centrality in collaborations that involve women has an additional positive impact. We also find that women inventors’ share in the pool of inventors is positively associated with innovation quality both at the county and regional levels. Furthermore, our findings indicate that in the network of women inventors, countries and regions that are in cohesive clusters (formed by repeated interactions between interconnected actors) show stronger innovation performance. Our study also highlights important nuances between country-level and region-level effects.

## Introduction

Economic geography literature has been pre-occupied with the analysis of factors that boost regional and country innovation, because of the importance of innovation for technological development and economic growth [1,2] While earlier studies mostly looked at the factors within regional and country borders such as human capital, education, innovation policy [3], more recent studies have argued that innovation does not happen exclusively within the borders of economic agents and started to analyze the phenomenon of social embeddedness of countries and regions that pertains to different types of linkages that connect countries and regions such as FDI flows [4] or trade linkages trade linkages [5,6] and their effects on innovation. Researchers found that, for instance, collaborations between subsidiaries and interactions between employees located in different countries facilitate the flows of knowledge and information between countries and those countries that are central in such flows have a positive effect on their innovation performance [7]. In a similar vein, scholars looked at the effects of connectedness between different regions via labor turnover and inter-firm linkages and found significant effects of connectedness on innovation of regional clusters [8].

While economic geography scholars looked at employee linkages, inter-firm linkages, trade linkages and their implications for country and regional innovation, much less attention has been devoted to collaborations between inventors and how they connect different regions and countries. At the same time, some studies argued that the invention process cannot be analyzed without paying attention to the interactions that occur among inventors [9].

Research on co-inventor networks has been quite prolific at the individual level [10–16]. While there exist different forms of innovation (product, organizational, marketing, etc.), researchers who have analyzed co-inventor networks have been particularly interested in patents and the impact collaborations have on inventors’ patent productivity. Scholars have been using patents to measure innovation because an invention must show an element of novelty, that is, some new characteristic that is not known in the body of existing knowledge in its technical field (https://www.wipo.int/patents/en/faq_patents.html), to be granted a patent. Additionally, patents must contain citations to other patents to acknowledge the state of the art. “These citations are understood to represent knowledge flows between inventors and have been widely used to study various economic and social aspects of innovation dynamics” [15].

There is evidence that having a more central position in collaboration networks has a positive impact on the number of patents an inventor obtains as well as the number of citations their patents receive [17]. However, our knowledge of the geographic distribution of collaboration networks is limited. We therefore do not know how different countries and regions are positioned in co-inventor networks, which countries and regions are more central, or whether a country’s or region’s position in co-inventor networks has any effect on its innovativeness, and whether the network effects are stronger at the country or regional level [16,18]. Another unanswered question is how differences in inventor attributes such as gender affect innovation performance. In this paper, we aim to contribute to the emerging literature on the economic geography of co-inventor networks and explore these questions with a particular focus on gender-related aspects in co-inventor networks and their effects on innovation performance at the country and regional level.

Many recent studies have been preoccupied with the gender gap in innovation, scientific research and patenting activities [19–24]. Journal publications’ findings are at times contradictory because some scholars find women are at a considerable disadvantage compared to men in terms of publishing in journals with a high impact factor and having their publications cited [25–27], whereas others find no significant differences between women and men, and some even find women in the field of management experience some benefits [28]. With regard to patents and innovation in corporate and industrial environments, there is general agreement in the literature that even though the proportion of women inventors is improving over time [29], R&D teams are dominated by men and men generate more patents [30–32]. Sugimoto et al. [31] also clearly show that, in every technological field, women are more likely to generate patents in academia than in corporate environments. Moreover, existing research shows that women tend to be less central in co-inventor networks, which negatively affects their innovation performance [33]. At the same time, emerging studies in gender literature have found that in terms of the quality of innovation, men and women innovate differently and having gender diversity in R&D teams extends the scope of team innovation [34,35]. Nevertheless, economic geography literature has yet to pay attention to gender-related aspects and their effects on innovation. We do not know whether women’s participation in patenting has a significant impact on a country’s or region’s innovation performance or whether countries and regions that are better positioned in women-led collaborative networks gain advantages in terms of their innovation productivity and the quality of their innovations. In this paper, in addition to analyzing the effect of the all-inventor collaborations on regional and country innovation, we also investigate the economic geography of patenting by women by analyzing their participation in patenting activity across regions and countries and by building country- and regional-level networks led by women inventors and analyzing their effects on innovation at the country and regional levels.

Recently, regional and national-level policymakers have started to realize the importance of strengthening collaborations across regions and countries for innovation and have started to give incentives to firms and inventors to engage in joint innovation projects. For example, the European Union developed Hoziron Research and Development program where calls for tenders specifically require inclusion of international partners. Understanding if the specific network and gender-aspects play a role in regional and country innovation, could make such policy making efforts more targeted and strategic.

The rest of the paper is structured as follows. The next section presents our literature review and our theoretical insights and propositions; the one thereafter presents our data, methodology and empirical analysis; and the final section includes a discussion of our findings, our conclusions, research limitations and areas for future research.

## Co-inventor network effects on the innovation performance of countries and regions

Innovation and technological development are important for economic growth [1,2]. The general literature argues that countries and regions that produce more patented innovations have considerable advantages as their economies are more diversified, more flexible, resilient and adaptable to various external shocks and economic downturns, and they can more easily nurture and grow new industries and new industrial segments [36].

As far as countries are concerned, the existing literature explores a variety of factors affecting a country’s innovativeness, from education level, innovation culture and specific policy initiatives [3] to its embeddedness in global structures and various linkages among countries [6]. The most popular linkages are trade linkages [5,6], foreign direct investment (FDI) flows [4,7] and country positioning in product space networks—an argument from recent economic complexity studies related to the positive impact of a country’s trade diversification and production of more complex products that have many linkages with other products [37]. Recent studies have also looked into immigration flows and their effects on innovation [38]. A common argument among these different strands of the literature is that a country’s innovativeness and economic performance are positively associated with its centrality in trade, FDI flows and other types of linkages. The reason behind this is that being central allows a country to have a competitive edge over more peripheral countries, be at the forefront of knowledge spillovers and have access to boundary-spanning knowledge, and more quickly adopt the latest technologies [6,39].

When it comes to regions, the main factors that drive innovation are region size, the availability of skilled labor, and local policies that support innovation [40]. At the same time, some scholars argue that the invention process cannot be well understood without paying attention to the social interactions that occur among inventors [9]. While research on co-inventor networks has mostly been conducted at the individual level, some works have started to look at the effects of inventor networks on regional innovation. For instance, research on the United States (US) urban system has shown that metropolitan regions with more local and non-local co-inventor linkages outperform cities whose economic agents are isolated [41]. In a similar vein, Fleming et al. [42] analyzed co-inventor networks between different regions and found that shorter path lengths and stronger connectedness correlates with increased innovation. Nevertheless, these studies have been carried out on relatively small data samples and in specific countries. We do not yet know the effect a region’s embeddedness in global co-inventor networks has on its innovation performance.

While there has been no country-level research on inventor networks in combination with linkages through trade or FDI and immigration flows, countries, like regions, are linked through co-inventor collaborations, and it is intuitive to think that these collaborations should influence country-level innovation dynamics too. Inventors who are central in collaboration networks have access to diversified ideas, knowledge and technologies that they can easily combine to produce new innovations [43,44]. As a result, regions and countries that are connected by many collaboration linkages have access to diverse knowledge, ideas and information and see their innovativeness get an additional boost. Therefore, it is logical to assume that countries and regions that are central in collaboration networks will have a competitive advantage over more peripheral countries and regions.

In addition to network centrality, there is another important network feature that is usually considered by network scholars—network clustering. Network clustering is different from network centrality because a cluster represents a subnetwork in which the focal node is directly linked to another node in the subnetwork [45]. An inventor who is located at the periphery of the network can still have a high clustering coefficient if he or she is located in a local network cluster with very dense linkages among its inventors. However, to be central in the overall network, an inventor must have far-reaching ties that connect him or her with many other inventors located in different network clusters [46]. The more general social network literature argues that clustering generates social capital, which encourages trust and support among cluster nodes [47]. The innovation literature argues that clustering is important for innovation. For example, Tahmooresnejad and Beaudry [17] found that having tight collaborative connections had important effects on innovation in academia in institutions where collaborative ties among inventors are dense. Studies of innovation in firms [48] discovered that firms that have tight collaborative connections among inventors are more innovative and more likely to develop and use new technologies. Additionally, the extensive literature on industrial clustering mentions the importance of social networking inside clusters and the positive effect it has on cluster innovation performance [8,49]. The country-level literature on trade and FDI networks argues that clustering is important for countries as it helps them build cohesive groups with resilient and trustworthy relationships and gain a critical mass and a reputation in the overall network. As a result, they attract more FDI and become more interesting for potential new trading partners [5]. Similarly, a country’s location in a dense co-inventor network cluster may help increase its reputation and send a signal to inventors from countries outside the cluster to start collaborating with its inventors. For example, Lim and Han’s study [50], found that there was strong collaboration between inventors from countries in East Asia, which gave them important critical mass in the network of all inventors and made them attractive to other inventors from outside the regional cluster. Clustering has not yet been explored in depth at the regional level, but Turkina and Oreshkin [16] provide a city-level example of clustering at work. They show that while Montreal cleantech inventors initially collaborated mostly with North American inventors, over time, they have welcomed more diverse collaborations and Montreal has become an internationally attractive hub—inventors from all over the globe have started to express a desire to collaborate with cleantech inventors in Montreal [16]. Linkages that formed between Montreal and other North American hubs were critical in improving Montreal’s innovation performance and, in the long run, helped to increase the number of global collaborations it has and to position it as a global hub.

Even though the existing literature indicates that there may be positive effects at the country and regional levels, it is important to analyze them separately, because in some environments regional-level dynamics may be different from country-level dynamics [51], especially in big countries like the United States or Canada, due to differences in regional innovation structures.

It is interesting to see if the effects are driven more by the country-level dynamics or the regional-level one.

The above discussion can be summarized by the following propositions:

***Proposition 1a***: *There is a positive association between a country’s centrality in the network of all inventors and its innovation performance*.
***Proposition 1b***: *There is a positive association between a region’s centrality in the network of all inventors and its innovation performance*.
***Proposition 2a***: *There is a positive association between a country’s position in a cohesive network cluster and its innovation performance*.
***Proposition 2b***: *There is a positive association between a region’s position in a cohesive network cluster and its innovation performance*.

## Gender-related aspects of co-inventor networks and their implications for regional and country innovation

As far as the gender-related aspects of co-inventor networks are concerned, while gender issues have not yet been explored in the context of country- and regional-level innovation studies, they have garnered significant interest among management and entrepreneurship researchers [52]. Increasing interest is being shown in women’s role in the labor force and how they contribute to economic development and economic growth. McKinsey Global Institute claimed that improving equality and enhancing the participation of women in the economy could increase annual GDP by $28 trillion in 2025—that is equivalent to the US’s and China’s current economies combined (a total of 95 countries were examined in this study to map gender quality indicators). Studies in management argue that increasing the share of women directors may yield better firm performance [53,54] because more diverse boards may generate different ideas and help to develop a better understanding of consumers and the market.

When it comes to innovation research, innovation studies largely lack a gender perspective [55]. While many studies try to evaluate whether or not there is a gender gap in science and innovation, very few explore the effects of women’s participation in inventions and women’s impact on innovation. Nählinder [56] and Blake and Hanson [57] highlight that men and women innovate differently, as women usually base their innovations largely on local needs with a view to social impact, whereas men produce more general innovations. A recent study in *Science* by Koning et al. [32] examines US biomedical patents and found that fewer women than men engage in commercial patenting and, as a result, there is a lack of breadth in inventions. The study found that when women are involved in biomedical inventions, their patents are more likely to focus on women’s health. The article claims that “the inventor gender gap is partially responsible for thousands of missing women-focused inventions since 1976.” Turner [34] showed that gender diversity improves R&D teams’ innovation performance, and in a similar vein, Diaz-Garcia et al. [35] demonstrated that gender diversity in R&D teams is positively related to radical innovation. Based on these pieces of evidence, it can be argued that countries and regions that have a higher share of women inventors in their inventor teams have broader, more diversified and more radical innovations, which results in stronger patenting activity.

As for the collaborative aspect, the existing research indicates that co-inventor collaboration has important effects on innovation [58]. Collaboration is critical for women, who are generally less central in collaboration networks and are underrepresented in commercial patenting [33]. Centrality in co-inventor networks enriches women with new ideas and knowledge and gives them important access to boundary-spanning knowledge that is often not available in their local environments. Additionally, existing literature in innovation indicates that due to the globalized nature of the world, solutions to local problems often come from knowledge about global issues (for instance, global trends in artificial intelligence, etc.) therefore even though women are more focused on their local situations, exposure to global collaborations and approaches can empower them to find better solutions to their challenges. Moreover, recent literature also indicates that the participation of women in inventors’ teams supports a faster career progress to becoming a prolific inventor and producing high quality innovations [59]. Thus, the participation of women in the innovation networking process is important for innovations produced by men too. Therefore, it can be assumed that countries and regions that are central in the network led by women inventors see an additional boost in their innovativeness.

In terms of the clustering aspect, women inventors usually have fewer collaborative relationships than men inventors. At the same time, they are also more likely to be in collaborative groups in which their co-authors are also linked [60]. Furthermore, the existing literature indicates that women inventors are more productive in cohesive collaborative groups than men inventors and tend to repeat their past collaborations [61,62]. Existing studies demonstrate similar trends in academic research output, as women co-author more often with the same people, and a higher fraction of their co-authors collaborate with each other [59]. The more general sociological literature argues that women feel more comfortable in connections in which there is a high level of trust and reciprocity [60] and are more process-oriented in social relationships and are more dependent on the social capital, whereas men are more result-oriented [63]. It is therefore logical to expect that a country’s or a region’s location in a cohesive cluster in the network of women inventors, where collaborations are dense and repeated, will be positively associated with its innovation performance.

***Proposition 3a***: *Countries that have a higher share of women inventors are more likely to be innovative*.
***Proposition 3b***: *Regions that have a higher share of women inventors are more likely to be innovative*.
***Proposition 4a***: *There is a positive association between a country’s centrality in the network of women inventors and its innovation performance*.
***Proposition 4b***: *There is a positive association between a region’s centrality in the network of women inventors and its innovation performance*.
***Proposition 5a***: *In the network of women inventors*, *countries that are in cohesive clusters show stronger innovation performance*.
***Proposition 5b***: *In the network of women inventors*, *regions that are in cohesive clusters show stronger innovation performance*.

## Data and methodology

The analysis presented in this paper uses data that was derived from OECD patent database. This database focuses on patents issued between 1978 and 2019 and their inventors, and contains 3,497,675 patents and 6,419,197 inventors from 235 countries and 5132 regions. Our analysis focuses on the co-patenting network that is represented by this data. We started by identifying the inventors’ gender, which is essential for our study since we aim to investigate the impact of characteristics of the network of women inventors at the country and regional levels. We used inventors’ names and gender recognition databases to determine inventors’ gender. Due to the various forms names take from one country to the next, we needed to clean up the inventors’ first names to improve the intelligence and quality of the matching process.

In our study, we used the World Intellectual Property Organization’s gender database developed by Martínez et al. [29]. To identify gender, we used the method provided by Hu et al. [64]. Their approach involves analyzing over 100 million first names and training a machine learning classification model based on the composition of name strings. Application of this model demonstrated a significant improvement in accuracy of gender prediction compared to other available models [64].

Using the classification of inventors’ patents, we observed that women inventors are most active in the IPC code categories “Chemistry; Metallurgy,” “Human Necessities,” and “Electricity,” while men inventors are most active in the “Chemistry; Metallurgy,” and “Performing Operations; Transporting” categories. Detailed category percentages are provided in Table B1 in Appendix B in S1 Appendix.

Afterwards, we constructed a network of all inventors and a network of women inventors using co-patenting links. A network link was assigned between two inventors if they had a joint patent. We constructed the networks based on source and target nodes, with source nodes being women inventors who collaborate with other (men or women) inventors. This study focuses on patents that have been granted. An application for a patent is a pending patent, and once it meets all the requirements for patentability, the patent is granted to the inventor. As a proxy measure of inventive capability, we restricted our sample to the most productive inventors and selected the inventors who had at least ten patents (we initially examined several scenarios ranging from 1 to 50 patents per inventor between 1978 and 2019. Restricting the number of patents considered made it possible to work with a proportion of women inventors to men inventors of approximately 8%. It was important for us to focus on prolific inventors and create a visual representation of the collaboration network over time, so we selected those inventors with at least 10 patents over the entire period considered). Building networks based on the most prolific inventors helped us to perform a more in-depth analysis with a geographical distribution of higher patenting productivity. Focusing on prolific inventors is justified in the literature [59] as they show more longevity and stronger innovation output, also patents of such inventors are more collaborative; therefore, such networks show better connectedness and are more feasible for the social network analysis. We assigned the patents to geographic locations using the inventors’ respective affiliation address.

### Network modeling at the country level

In our collaboration networks, individual countries act as nodes and a network link is assigned between two countries when a joint patent has inventors located in those countries.

We investigate first a dynamic view of countries including all prolific inventors and then one of countries including only prolific women inventors. The network of all inventors includes 47 countries, and the one of women inventors includes 35 countries.

To better measure collaboration in this study, we construct two versions of each network. The first is a static version, representing the countries and co-patenting links between countries for the entire time period. It gives us insight to visualize the network. The second is a dynamic version that represents 5-year intervals in each year by considering all the links in the four years prior to a specific year and that year to calculate annual network characteristics. This version helps us to describe the implications of changing network structures over time and helps us to investigate the behavior of network metrics over time. Therefore, we use the five-year interval network indicator to examine the impact of network positioning on patenting performance and citation impact. The network indicator is organized as a time series for each year over a forty-year period.

**Fig 1:**
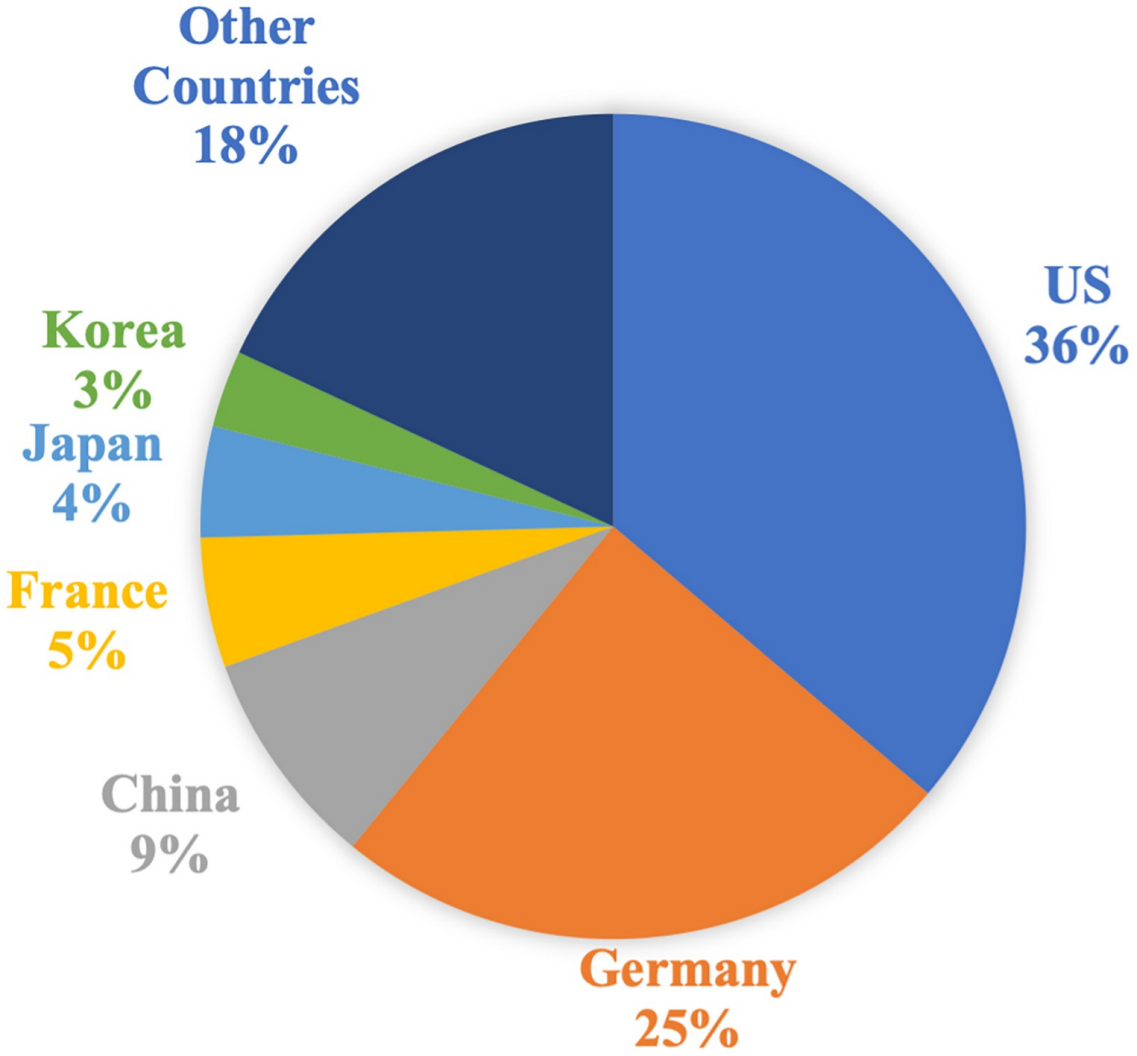
Distribution of countries with the highest number of women inventors, 1978–2019.

Fig 1 presents the distribution of countries with the highest number of women inventors over the entire period of our analysis. The US and Germany dominate the graph and are clearly a step ahead, followed by China, France, Japan and Korea. Other countries have little weight compared to the US and Germany.

**Fig 2:**
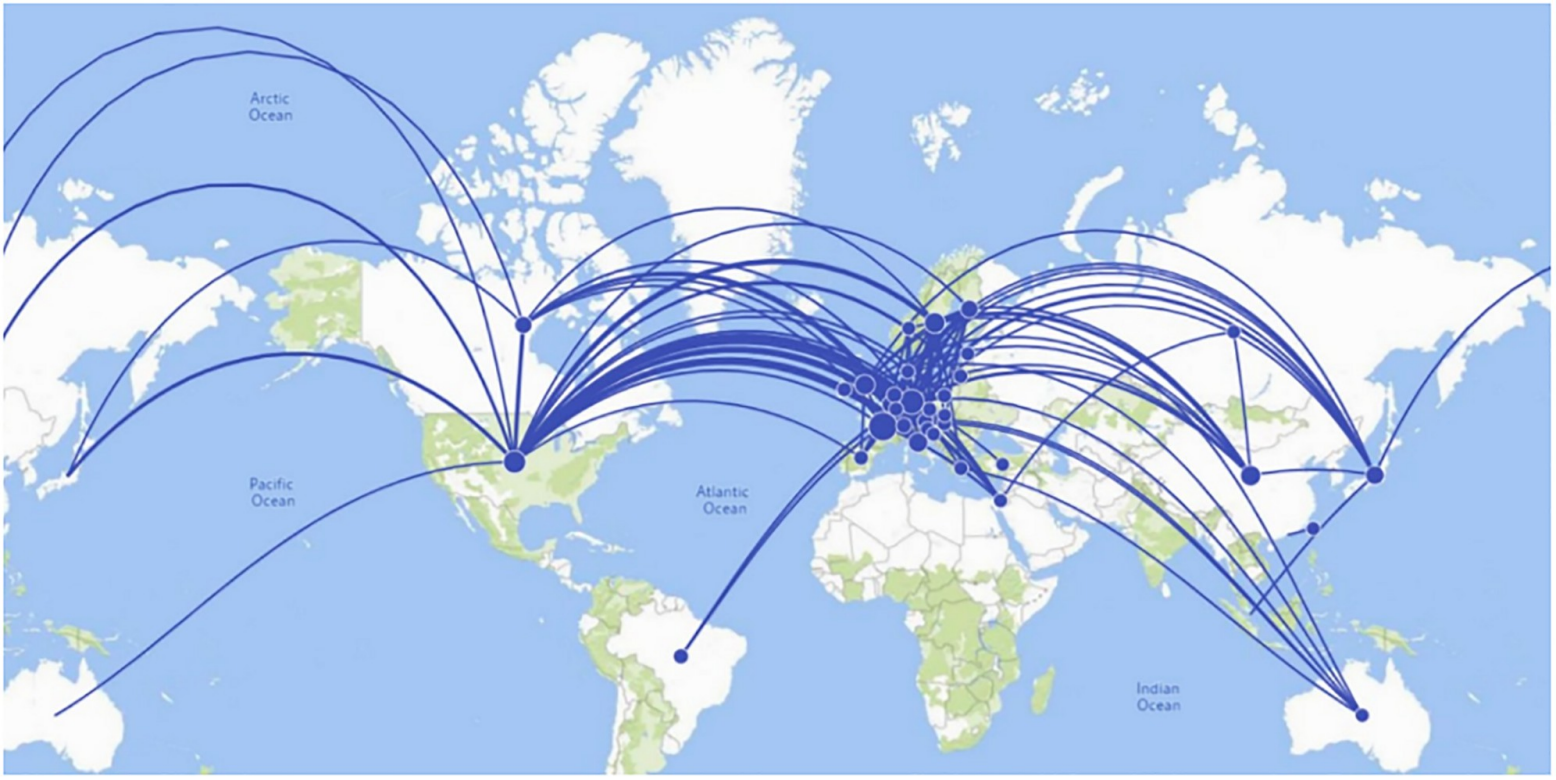
Co-patenting network of all inventors at the country level (Geographical mapping).

**Fig 3:**
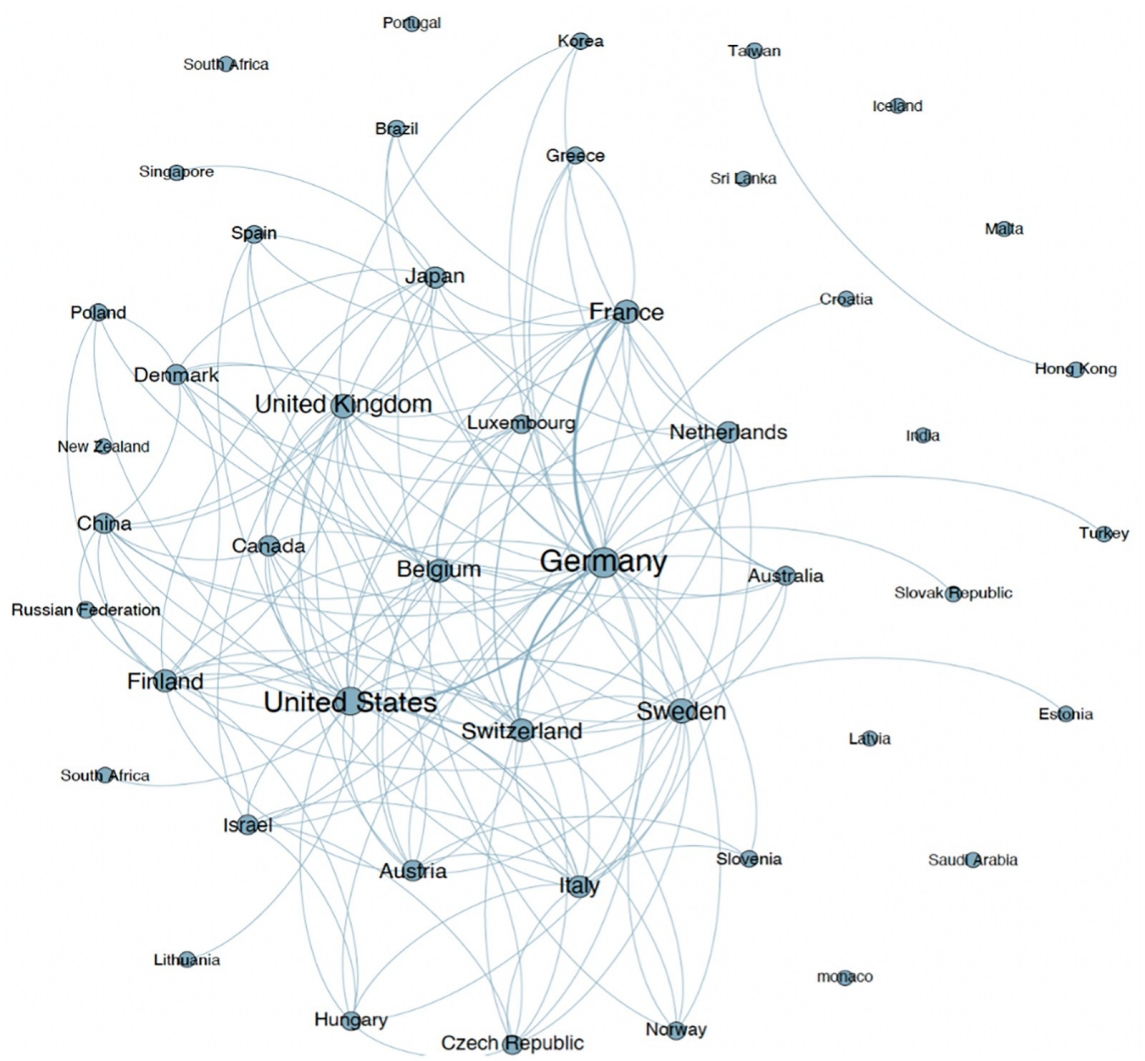
Co-patenting network of all inventors at the country level (by country).

**Fig 4:**
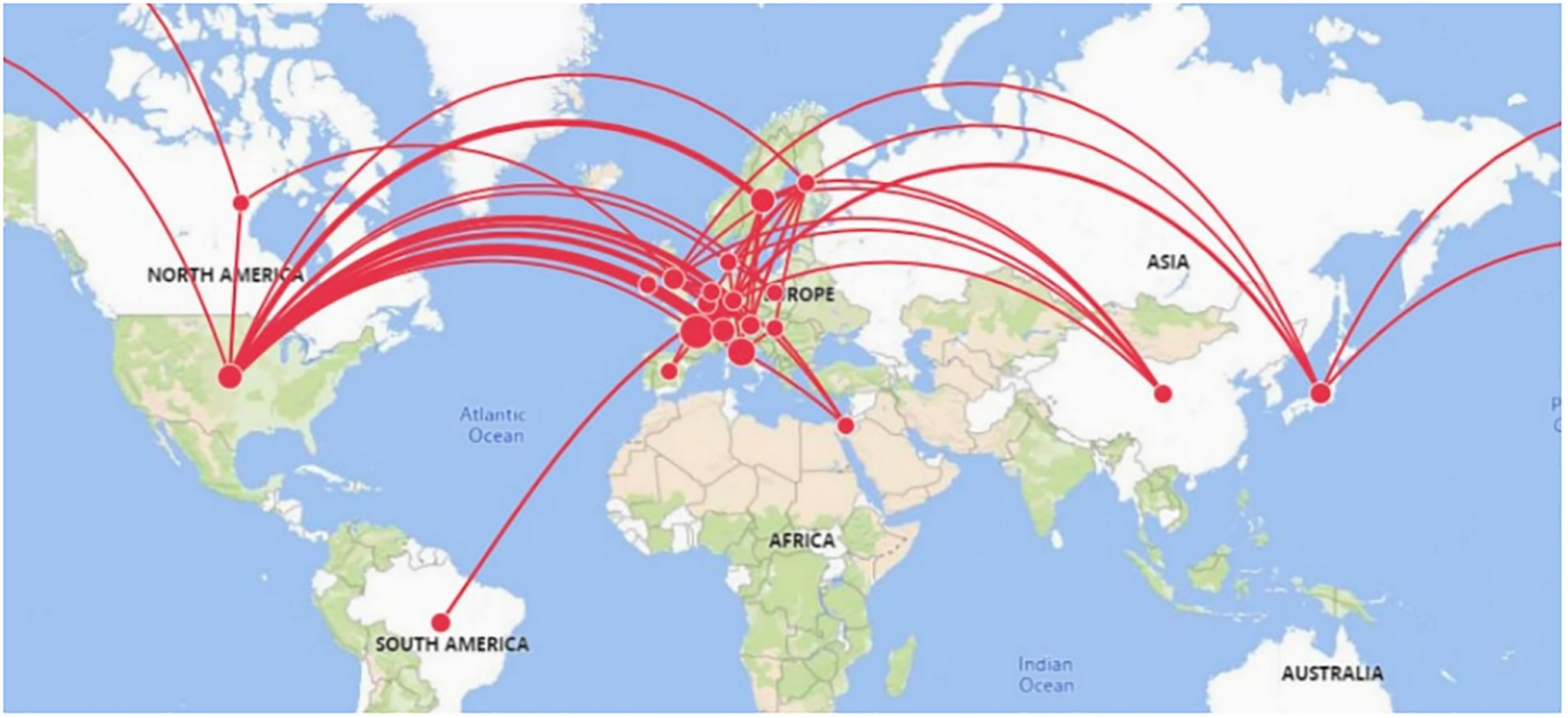
Co-patenting network of women inventors at the country level (Geographical mapping).

**Fig 5:**
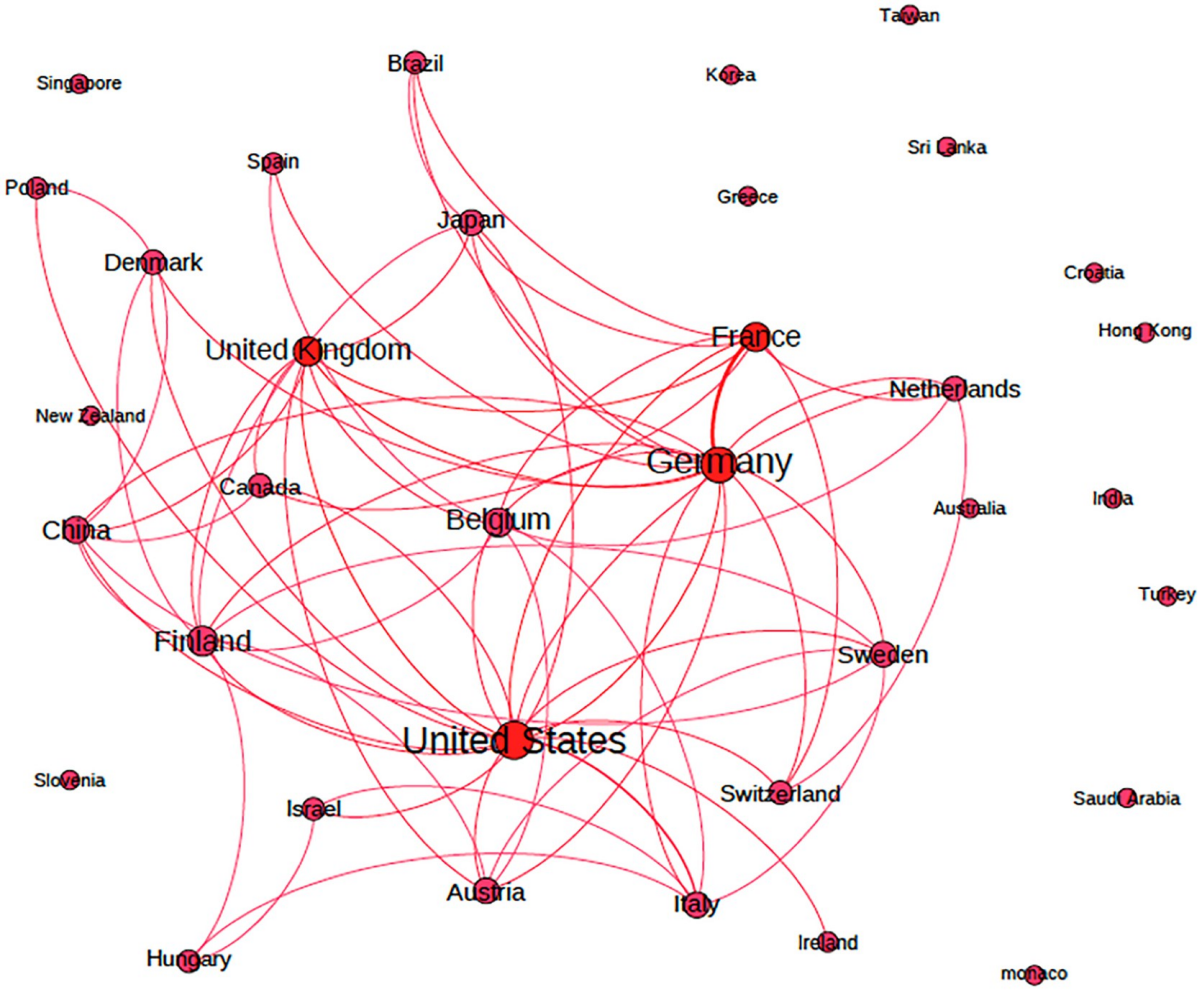
Co-patenting network of women inventors at the country level (by country).

We show the country-level networks in Figs 2 to 5. Figs 2 and 3 present the network for all inventors (men and women), and Figs 4 and 5 present the network considering only women inventors. The thickness of the lines represents the weight of the co-links between the countries. The number of links that appear in these figures indicates that the most active countries are Germany and the US. When we examine the weight of the links in the network, we find that inventors in Germany collaborate more with inventors in France, Switzerland and the United States than those in the other countries. The five co-links having the highest number of weighted links in each network (of all inventors and of only women inventors) are set out in Table 1 (note that the networks are undirected).

**Table 1:** The top five weighted links of each network.

Source Country	Target Country	Weighted Links
***Network of All Inventors***		
Germany	France	1,685
Switzerland	Germany	1,189
Germany	United States	827
Switzerland	France	579
United States	Italy	564
***Network of Women Inventors***		
Germany	France	442
United States	Italy	297
United States	Sweden	136
Germany	United States	116
Germany	Switzerland	99

The strongest links in the network of women inventors are between Germany and France and between the United States and Italy.

**Fig 6:**
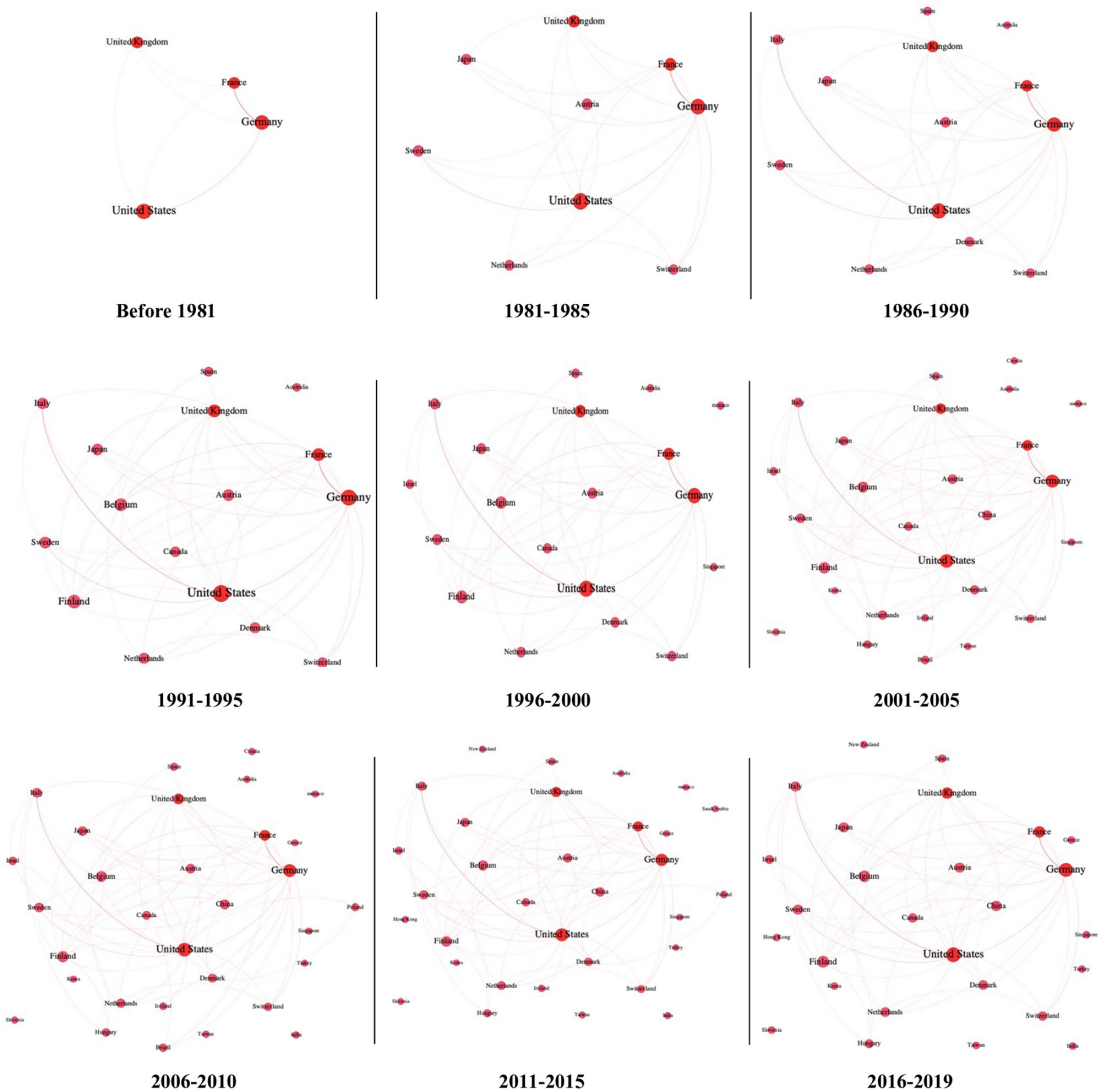
The evolution of the network of women inventors at the country level over time.

We then divide the country-level co-patenting network of women inventors into nine intervals in Fig 6 to clearly visualize links over time.

The visualization reveals some interesting features that are easier to see than they are to determine from the indicators. For instance, only four countries (the United States, the United Kingdom, Germany and France) are connected to one another in the early years of the time period studied. Over time, women inventors in many countries such as Sweden, Swithzerland, Austria and Japan appear to engage in international collaborations. Also, the number of collaborations between countries increases each year. It is remarkable that the co-links between women inventors in Italy and the United States and in Germany and France have grown significantly over the intervals.

In Table 2, we compare the network centrality measures of the 10 countries that are the most central in the women inventor network over the entire period (variable description section presents the variables and gives a detailed description of the four centrality measures. In sum, degree centrality reflects the number of direct connections, closeness centrality reflects the overall centrality of the node in the network including direct and indirect connections, eigenvector centrality reflects connectedness to the highly connected nodes, and betweenness centrality reflects how often the node connects otherwise disconnected nodes). This table shows that the United States and Germany have the most connections with other countries when it comes to women inventors. Some countries like the United Kingdom, Belgium and France, which have the same degree centrality, have different intermediary roles because we see Belgium has the highest betweenness centrality value of these three countries. Another interesting observation is that when we compare the betweenness centrality of Italy and Sweden we find that women inventors in Italy connect with more inventors from different countries than those in Sweden do. The US, Germany and Finland have the highest closeness centrality scores, meaning that these countries can rapidly develop collaborations because their inventors have the easiest access to inventors in other countries in the network.

**Table 2:** The 10 most central countries in the women inventor network.

Country	Degree Centrality	Eigenvector Centrality	Closeness Centrality	Betweenness Centrality
United States	17	1.0000	0.8696	0.1202
Germany	15	0.9573	0.8000	0.0666
Finland	10	0.7271	0.6667	0.0253
United Kingdom	9	0.7156	0.6452	0.0075
Belgium	9	0.6771	0.6452	0.0162
France	9	0.6326	0.6250	0.0141
China	7	0.5600	0.6061	0.0047
Sweden	6	0.5063	0.5882	0.0018
Italy	6	0.4225	0.5882	0.0126

Our study sheds light on the links that exist between countries in which women inventors are active players when it comes to advancing technology. We note that the United States and Germany are the top two countries in the women inventor network and that they are closely connected to France, Italy, Sweden, Switzerland and one another. Moreover, this paper identifies developed countries, such as Belgium, Finland, and Denmark, that have established links with top developed countries that play a significant role in the network of women inventors. In addition, we acknowledge the presence of some developing countries such as India, Turkey, Taiwan, Saudi Arabia, Singapore, and Sri Lanka in the women inventor network (as indicated in Fig 3), but we cannot observe their international collaborations since their inventions are developed mostly within the country. Our findings also revealed that not all developed countries have internationally prolific women inventors. For example, Korean, Hong Kong, Slovenian, and Greek women inventors seem to prefer to collaborate with inventors in their own countries and do not collaborate with peers beyond those borders.

**Fig 7:**
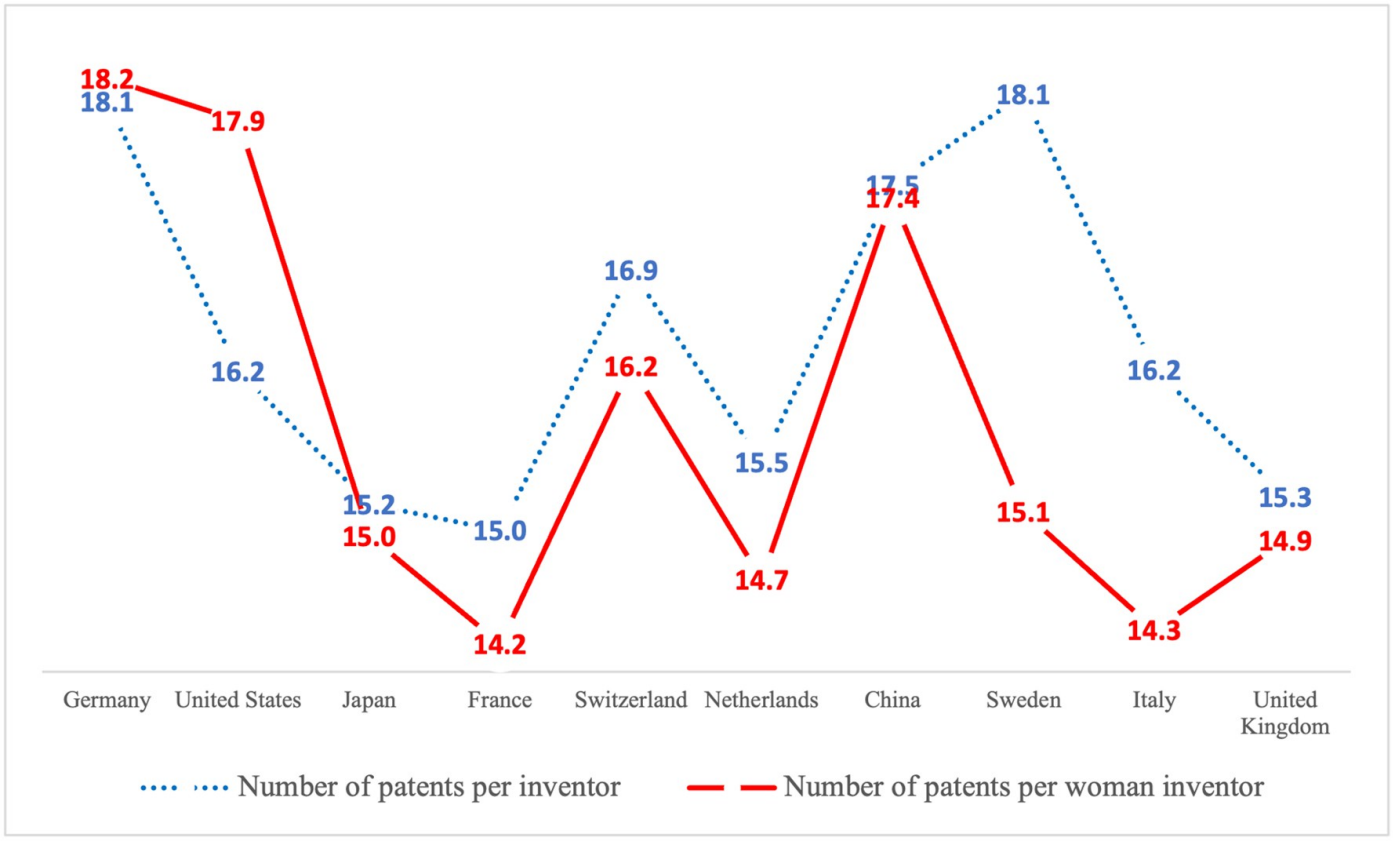
The number of patents per inventor and per woman inventor in the 10 countries having the highest number of inventors.

In Fig 7, we plot the number of patents per inventor and the number of patents co-invented by women inventors per woman inventor in the 10 countries having the highest number of inventors. These values enable us to better compare the patenting activity of women inventors in select countries. As we see in the Fig 7, the number of patents per woman inventor is higher than the number of patents per inventor in Germany and the United States, and the two values are close in Japan and China. It is interesting to see that when we consider the 10 countries having the highest number of specifically women inventors (this graph is presented in Appendix C in S1 Appendix), Canada, Finland and Korea make the list, and the United Kingdom doesn’t. Moreover, we find that the number of patents per woman inventor is higher than the number of patents per inventor in Canada and Finland.

### Network modeling at the regional level

We are also interested in studying collaborations at the regional level. We construct one regional network of all inventors and one of women inventors. In each one, the nodes are regions, and a network link is assigned between two regions when a joint patent has inventors located in those regions. Our regions reflect their respective OECD database definition within national borders and, in most cases, correspond to administrative regions. The REGPAT’s regional breakdowns are taken from two sources: the 2013 version of the Nomenclature of Territorial Units for Statistics (NUTS, 2013 Eurostat) for European countries (NUTS3), and the OECD’s Territorial Level 3 (TL3) for other countries.

Our analysis reveals that regions are key organizing units for innovation in each country and are at the core of the invention process. Table 3 shows four centrality measures for most connected regions from different countries in all inventor networks. At first glance, we see that regions that host big global cities, such as Berlin, Montreal GTA, Beijin, etc. account for a considerable share of innovations. We also see interesting trends that while German regions such as Berlin have the highest eigenvector and closeness centralities, which indicates their overall connectedness in the networks, US regions such as Santa Clara County have higher betweenness centrality indicating that they are important intermediaries in the global innovation network. While regions from developing countries (except for China) have low centralities, we see that regions like Sao Paolo (Brazil) and Tekirdag (Turkey) also participate in the global collaboration network.

**Table 3:** Top 10 most connected regions from different countries in all inventor networks.

Region	Country	Degree Centrality	Eigenvector Centrality	Closeness Centrality	Betweenness Centrality
Berlin	Germany	119	0.9848	0.4048	0.0244
Santa Clara County, CA	US	79	0.2390	0.4013	0.0422
Rhône	France	49	0.3127	0.3573	0.0165
Basel-Landschaft	Switzerland	44	0.3670	0.3637	0.0065
Helsinki-Uusimaa	Finland	42	0.1661	0.3693	0.0109
Beijing	China	35	0.1577	0.3664	0.0053
Leuven	Belgium	33	0.1660	0.3422	0.0057
Skåne County	Sweden	31	0.1747	0.3561	0.0033
Montreal (Communauté-Urbaine-de-Montréal), QC	Canada	29	0.1014	0.3388	0.0026
Hampshire County	England	26	0.1872	0.3410	0.0052
São Paulo	Brazil	7	0.0772	0.3027	0
Tekirdag	Turkey	1	0.0020	0.2258	0
Uttar Pradesh	India	1	0.0003	0.01	0

Table 4 presents most connected regions in the women inventor network. In general, German regions clearly dominate women inventors’ collaborations. At the same time, regions in the US, as well as in Finland and France have higher betweenness centrality, indicating that they play the role of intermediaries in the network and connect many otherwise disconnected regions. As far as the developing countries are concerned, we see that while Sao Paolo is also a participant of the global women-led network, Turkish Tekirdag is not connected in the women inventor network.

**Table 4:** Top 10 most connected regions from different countries in women inventor networks.

Region	Country	Degree Centrality	Eigenvector Centrality	Closeness Centrality	Betweenness Centrality
Düsseldorf kreisfreie stadt	Germany	44	1.000	0.316	0.031
San Diego County	US	29	0.188	0.302	0.044
Helsinki-Uusimaa	Finland	24	0.171	0.316	0.061
Rhône	France	18	0.450	0.309	0.041
Beijing	China	11	0.134	0.296	0.009
Communauté Urbaine de Montréal QC	Canada	10	0.033	0.240	0.013
Basel-Stadt	Switzerland	7	0.036	0.239	0.002
Vienna	Austria	6	0.064	0.257	0.005
Leuven	Belgium	5	0.019	0.231	0.004
Ibaraki	Japan	5	0.130	0.251	0.000
São Paulo	Brazil	4	0.1228	0.2513	0.0000
Uttar Pradesh	India	1	0.0007	0.01	0.0000

### Description of the variables

Network measures have been widely used to explore the impact of collaborations. In this study, a network analysis that takes co-invention links between countries/regions into consideration provides additional insights into collaborations at the country and regional levels. Complex network indicators are an effective tool to analyze global and regional co-patenting structures from a complex network perspective. A variety of network and inventor characteristics are therefore introduced to better highlight the influence of network positioning on patenting activity.

*NumPatCtry*_*t*_/*NumPatRegion*_*t*_, which represent the number of patents a country/region obtains in a given year, and *NumCitCtry*_*t*_/*NumCitRegion*_*t*_, which represent the number of citations a country’s/region’s patents receive in a given year, are used as our dependent variables in the country/regional overall inventor networks, respectively. Similarly, we use the variables *NumFemPatCtry*_*t*_/*NumFemPatRegion*_*t*_, which represent the number of patents a country/region obtains in a given year that include at least one woman inventor, and *NumFemCitCtry*_*t*_/*NumFemCitRegion*_*t*_, which represent the number of citations a country’s/region’s patents that include at least one woman inventor receive in a given year, as our dependent variables in the country/regional women inventor networks, respectively.

Degree centrality is one of the network measures that are included in the models. It reflects the number of direct connections a county or a region has. Four degree centrality variables are taken into account—*CtryDegCentrality*_*t*_ and *CtryDegFemCentrality*_*t*_ at country level, and *RegionDegCentrality*_*t*_ and *RegionDegFemCentrality*_*t*_ at region level.

The eigenvector centrality thesis states that a country/region is important if it is linked to other highly connected countries/regions by co-invention links. When it comes to eigenvector centrality, having many links does not necessarily mean a high eigenvector centrality value as the eigenvector centrality of all the countries/regions to which a given country/region is linked may be low or zero). The variables we use are *CtryEigenvector*_*t*_/*RegionEigenvector*_*t*_ for the country/regional networks of all inventors, respectively, and *CtryFemEigenvector*_*t*_/*RegionFemEigenvector*_*t*_ for the country/regional networks of specifically women inventors, respectively.

A country’s/region’s importance in a network can be determined using betweenness centrality, which is widely used in network theory and measures a country’s/region’s bridging nature and intermediary position in a co-invention network. Betweenness centrality takes into account the number of shortest paths from one country/region to another that pass through the country/region in question. The countries/regions with higher betweenness centrality are able to exert more control over the network, since more information is passed through them. A country/region in our network can be considered well connected if it is located on as many shortest paths between two other countries/regions as possible. The assumption behind using betweenness centrality is that two countries/regions that are not directly connected are dependent on the other countries/regions in the network that are positioned between them to collaborate with one another.

The betweenness centrality variables that we employ in our models are *CtryBetweenness*_*t*_/ *RegionBetweenness*_*t*_ for the country/regional networks of all inventors, respectively, and *CtryFemBetweenness*_*t*_*/RegionFemBetweenness*_*t*_ for the country/regional networks of specifically women inventors, respectively.

Another network measure that uses geodesic distance in the network is closeness centrality. A country/region with a high closeness centrality is able to make contact with other countries/regions easily. Closeness centrality is a way of detecting countries/regions that are able to spread information through a co-invention network very efficiently and measures the average shortest distance between the nodes in a network. If a country/region can reach others easily, it can spread knowledge easily, even to countries/regions that are not directly connected to it. The closeness centrality variables that we use in our study are *CtryCloseness*_*t*_*/RegionCloseness*_*t*_ for the country/regional networks of all inventors, respectively, and *CtryFemCloseness*_*t*_*/ RegionFemCloseness*_*t*_ for the country/regional networks of women inventors.

Our last network measure is the clustering coefficient. It calculates the interconnectedness of a country’s/region’s neighbors in the network. More specifically, it is a measure of density; when connections are dense, the clustering coefficient is high. The clustering coefficient variables used in this analysis—*CtryClustering*_*t*_/*RegionClustering*_*t*_ for the country/regional networks of all inventors, respectively, and *CtryFemClustering*_*t*_/*RegionFemClustering*_*t*_ for the country/regional networks of women inventors, respectively—measure the probability that any two neighbors of a specific country/region are also connected to each other.

Network measures are calculated over a five-year period for each country/region each year, and all network variables are lagged by one year in the models. Hence, we deal with the panel data in our model in the form of network variables, and the related data is collected for each country/region each year.

It is also necessary to consider some characteristics related to co-inventors as control variables for a more systematic analysis. The number of co-inventors involved in a patent represents a group of collaborations that might affect patenting productivity and the patent’s citation impact. We compute the total number of patent co-inventors in a country and region in a given year (*NumInventors*_*t*_*)*. This variable helps us to understand the potential impact of relationships between co-inventors. The one variable that allows us to examine whether the time between an inventor’s or a country’s /region’s first and last patenting activities has had a positive effect on average in terms of patenting productivity and impact is the proportion of patenting activity time to the number of patents averaged at the country and region level (*PropYearPat*_*t*_). Additionally, the number of patents withdrawn in a country and region in a year (*PropWithdrawalPat*_*t*_) can be a proxy of lower productivity and requires attention.

Inventor share is different in a co-invention relationship, and we take average inventor share into account in our model as a control variable. We computationally generate a variable (*ShareInventor*_*t*_) based on each inventor’s share in each of their patents and then calculate the average for all the patents in which they are listed as a co-inventor averaged at the country and regional levels. One may also consider the effect of the share of women inventors among co-inventors. To test this effect, we include the proportion of the number of women co-inventors to all co-inventors averaged at the country and regional levels for all the inventors that are affiliated with a country /region (*ShareWomen*_*t*_).

This study also considers the diversification of co-inventors to evaluate the impact of being involved more in national or international collaborations. Some patents have a relatively more diverse group of co-inventors (regions and countries), which reflects the extent of co-inventors’ connections that lead them to quickly spread knowledge internationally and regionally. By using the *ShareCountry*_*t*_ variable, we are able to examine whether the relationship between co-inventors from different economies can affect the productivity and citation impact of the output at the international scale. All the variables utilized in this study are defined in Table 5.

**Table 5:** Definition of the variables.

Variable	Definition
***Dependent Variables***	
*NumPatCtry*_*t*_	The total number of patents a country obtains in a given year
*NumCitCtry*_*t*_	The total number of 3-year forward citations a country’s patents receive in a given year
*NumFemPatCtry*_*t*_	The total number of patents a country obtains in a given year that include at least one woman inventor
*NumFemCitCtry*_*t*_	The total number of 3-year forward citations a country’s patents that include at least one woman inventor receive in a given year
*NumPatRegion*_*t*_	The total number of patents a region obtains in a given year
*NumCitRegion*_*t*_	The total number of 3-year forward citations a region’s patents receive in a given year
*NumFemPatRegion*_*t*_	The total number of patents a region obtains in a given year that include at least one woman inventor
*NumFemCitRegion*_*t*_	The total number of 3-year forward citations a region’s patents that include at least one woman inventor receive in a given year
***Control Variables***	
*NumInventors*_*t*_	The total number of inventors in a country in a given year
*ShareInventor*_*t*_	The average inventor share of all the inventors in a country in a given year
*PropYearPat*_*t*_	The proportion of the number of years that a country has been active in terms of patenting activity, which denotes the first year in which it obtained a patent and defines the time by taking the number of active years from the first patenting year and dividing it by the total number of patents in a given year averaged at the country level
*PropWithdrawalPat*_*t*_	The proportion of the total number of patents withdrawn divided by the total number of patents obtained by a country in a given year
*ShareWomen*_*t*_	The average proportion of the number of women inventors to all inventors of a specific patent, averaged at a country level (Our analysis considers the gender count variable, which indicates whether a patent was co-invented by one gender (men or women) or both genders (men and women), as well as the *ShareWomen*_*t*_ variable. We include only one of these variables, *ShareWomen*_*t*_, in our analysis since the gender count and *ShareWomen*_*t*_ are highly correlated.
*ShareCountry*_*t*_	The average number of countries that a country’s inventors who patent are affiliated with in a given year
*GrossDomesticProduct*	A time-varying control variable taken from the OECD database for each year between 1970 and 2019
*Dummy_year*	A dummy variable to account for any time intervals in a scholar’s performance
***Network Variables***	
***Country level***	
*CtryDegCentrality*_*t*_	A country’s degree centrality in a given year in the co-patenting network of all inventors (a one-year lag is used in the model)
*CtryEigenvector*_*t*_	A country’s eigenvector centrality in a given year in the co-patenting network of all inventors (a one-year lag is used in the model)
*CtryCloseness*_*t*_	A country’s closeness centrality in a given year in the co-patenting network of all inventors (a one-year lag is used in the model)
*CtryBetweenness*_*t*_	A country’s betweenness centrality in a given year in the co-patenting network of all inventors (a one-year lag is used in the model)
*CtryClustering*_*t*_	A country’s clustering coefficient in a given year in the co-patenting network of all inventors (a one-year lag is used in the model)
*CtryFemDegCentrality*_*t*_	A country’s degree centrality in a given year in the co-patenting network of women inventors (a one-year lag is used in the model)
*CtryFemEigenvector*_*t*_	A country’s eigenvector centrality in a given year in the co-patenting network of women inventors (a one-year lag is used in the model)
*CtryFemCloseness*_*t*_	A country’s closeness centrality in a given year in the co-patenting network of women inventors (a one-year lag is used in the model)
*CtryFemBetweenness*_*t*_	A country’s betweenness centrality in a given year in the co-patenting network of women inventors (a one-year lag is used in the model)
*CtryFemClustering*_*t*_	A country’s clustering coefficient in a given year in the co-patenting network of women inventors (a one-year lag is used in the model)
***Regional level***	
*RegionDegCentrality*_*t*_	A region’s degree centrality in a given year in the co-patenting network of all inventors (a one-year lag is used in the model)
*RegionEigenvector*_*t*_	A region’s eigenvector centrality in a given year in the co-patenting network of all inventors (a one-year lag is used in the model)
*RegionCloseness*_*t*_	A region’s closeness centrality in a given year in the co-patenting network of all inventors (a one-year lag is used in the model)
*RegionBetweenness*_*t*_	A region’s betweenness centrality in a given year in the co-patenting network of all inventors (a one-year lag is used in the model)
*RegionClustering*_*t*_	A region’s clustering coefficient in a given year in the co-patenting network of all inventors (a one-year lag is used in the model)
*RegionFemDegCentrality*_*t*_	A region’s degree centrality in a given year in the co-patenting network of women inventors (a one-year lag is used in the model)
*RegionFemEigenvector*_*t*_	A region’s eigenvector centrality in a given year in the co-patenting network of women inventors (a one-year lag is used in the model)
*RegionFemCloseness*_*t*_	A region’s closeness centrality in a given year in the co-patenting network of women inventors (a one-year lag is used in the model)
*RegionFemBetweenness*_*t*_	A region’s betweenness centrality in a given year in the co-patenting network of women inventors (a one-year lag is used in the model)
*RegionFemClustering*_*t*_	A region’s clustering coefficient in a given year in the co-patenting network of women inventors (a one-year lag is used in the model)

## Discussion of the results

We conducted negative binomial regressions to determine whether the network measures influence the number of patents and the citation impact of patents a country has and understand the network positioning that may affect a country’s patenting activity. The fixed effects model is used in this study to help produce consistent coefficient estimates (the statistic estimated for the Hausman test was used to determine which model was most appropriate to estimate (the Hausman test verifies whether there is correlation between individual effects and other regressors under the null hypothesis). Also, the models were estimated for the potential existence of serial correlation and heteroskedasticity).

Tables 6 and 7 present the results for the number of patents and the number of patent citations at the country level, respectively, and Tables 8 and 9 present the results for the number of patents and the number of patent citations at the region level, respectively. The dependent variables were regressed on the country-level network measures and the average inventor characteristics for the countries in Tables 6 and 7, respectively, and on the regional-level network measures and the average inventor characteristics for the regions in Tables 8 and 9, respectively. All network measures with the exception of the clustering coefficient (therefore, *Degree*, *Eigenvector*, *Closeness* and *Betweenness*) are strongly and very positively associated with the number of patents and the number of patent citations received. Our results also provide insights into collaboration between countries. A country’s degree centrality is a basis for the degree of connections in patenting activities in that country. The importance of a country’s betweenness centrality in an innovation network confirms that when a country is frequently positioned as a connector along the shortest paths between pairs of other countries, the patenting performance of the inventors in that country is enhanced. Closeness centrality suggests that a country that has a lower sum of the geodesic distances to reach every other country in an innovation network can efficiently develop collaborations with those other countries and increase its patenting performance and impact. The eigenvector centrality captures the countries that are connected to highly central countries: the higher a country’s eigenvector centrality in the innovation network is, the higher its innovation performance is.

**Table 6:** The regression results of the impact of country-level network measures on the number of patents.

*Variables*	Models									
*NumPatCtry*_*t*_	(1)		(2)		(3)		(4)		(5)	
*CtryDegCentrality*_*t-1*_	0.910	***								
	(0.135)									
*CtryEigenvector*_*t-1*_			0.260	***						
			(0.046)							
*CtryCloseness*_*t-1*_					2.878	***				
					(0.507)					
*CtryBetweenness*_*t-1*_							0.068	***		
							(0.013)			
*CtryClustering*_*t-1*_									0.252	*
									(0.103)	
*NumInventors*_*t*_	1.076	*	1.340	**	1.416	**	1.000	*	1.500	**
	(0.565)		(0.675)		(0.680)		(0.601)		(0.739)	
*PropYearPat*_*t*_	-26.46	***	-29.512	***	-29.118	***	-25.021	***	-30.762	
	(4.680)		(6.345)		(6.249)		(5.985)		(7.098)	
*ShareWomen*_*t*_	-0.188		-1.856		-2.469		-0.893		-3.833	
	(1.554)		(1.861)		(2.070)		(1.928)		(2.293)	
*GrossDomesticProduct*_*t*_	0.486	***	0.562	***	0.581	***	0.584	***	0.674	
	(0.077)		(0.104)		(0.113)		(0.091)		(0.143)	
*Dummy_year*	Yes		Yes		Yes		Yes		Yes	
*Constant*	-2.491	***	-4.424	***	-9.778	***	-4.011	***	-5.604	***
	0.8522		(1.348)		(2.059)		(1.075)		(1.875)	
*lnalpha_constant*	-0.493	***	-0.285		-0.227		-0.364	**	-0.134	
	(0.170)		(0.181)		(0.185)		(0.172)		(0.197)	
*Nb of observations*	771		771		771		771		771	
*Nb of groups*	32		32		32		32		32	
*R* ^*2*^ *_pseudo*	0.1376		0.1205		0.1176		0.1274		0.1082	
*Loglikelihood*	-4712		-4806		-4893		-4768		-4873	

Note 1:

***, ** and * show significance at the 1%, 5% and 10% levels, respectively, and standard errors are presented in parentheses.

**Table 7:** The regression results of the impact of country-level network measures on the number of citations.

*Variables*	Models									
*NumCitCtry*_*t*_	(1)		(2)		(3)		(4)		(5)	
*CtryDegCentrality*_*t-1*_	0.516	***								
	(0.158)									
*CtryEigenvector*_*t-1*_			0.145	***						
			(0.056)							
*CtryCloseness*_*t-1*_					1.632	***				
					(0.566)					
*CtryBetweenness*_*t-1*_							0.044	***		
							(0.012)			
*CtryClustering*_*t-1*_									0.038	
									(0.096)	
*NumInventors*_*t*_	2.258	***	2.436	***	2.398	***	2.008	**	2.501	**
	(0.832)		(0.882)		(0.923)		(0.780)		(0.992)	
*PropYearPat*_*t*_	-35.53	***	-38.127	***	-38.102	***	-34.576	***	-39.478	***
	(5.317)		(5.726)		(5.795)		(5.477)		(6.200)	
*ShareWomen*_*t*_	4.922	*	4.010*		3.125		3.127		1.560	
	(2.660)		(2.661)		(2.528)		(2.627)		(2.876)	
*GrossDomesticProduct*_*t*_	0.559	***	0.591	***	0.607	***	0.606	***	0.648	***
	(0.103)		(0.109)		(0.111)		(0.100)		(0.114)	
*Dummy_year*	Yes		Yes		Yes		Yes		Yes	
*Constant*	-9.073	***	-10.068	***	-13.117	***	-9.541	***	-10.523	***
	(1.533)		(1.670)		(2.010)		(1.445)		(1.979)	
*lnalpha_constant*	0.151		0.182		0.201		0.151		0.220	
	(0.130)		(0.134)		(0.131)		(0.132)		(0.141)	
*Nb of observations*	771		771		771		771		771	
*Nb of groups*	32		32		32		32		32	
*R* ^*2*^ *_pseudo*	0.112		0.109		0.109		0.112		0.106	
*Loglikelihood*	9328		9356		9491		9328		9392	

Note 1:

***, ** and * show significance at the 1%, 5% and 10% levels, respectively, and standard errors are presented in parentheses.

**Table 8:** The regression results of the impact of regional-level network measures on the number of patents.

*Variables*	Models									
*NumPatRegion*_*t*_	(1)		(2)		(3)		(4)		(5)	
*RegionDegCentrality*_*t-1*_	0.488	***								
	(0.034)									
*RegionEigenvector*_*t-1*_			0.029	**						
			(0.007)							
*RegionCloseness*_*t-1*_					1.446	**				
					(0.359)					
*RegionBetweenness*_*t-1*_							0.030	**		
							(0.005)			
*RegionClustering*_*t-1*_									-0.232	
									(0.035)	
*NumInventors*_*t*_	1.337	***	1.130	***	1.165	***	1.145	***	1.0326	***
	(0.116)		(0.148)		(0.152)		(0.144)		(0.149)	
*PropYearPat*_*t*_	-3.765	***	-6.870	***	-6.751	***	-7.922	***	-9.543	***
	(0.314)		(0.264)		(0.276)		(0.342)		(0.288)	
*PropWithdrawalPat*_*t*_	-0.191		-0.022		-0.0364	*	-0.0915	*	-0.0334	*
	(0.277)		(0.302)		(0.305)		(0.297)		(0.299)	
*ShareWomen*_*t*_	1.694		1.932		1.9428		1.8736		1.9145	
	(0.071)		(0.079)		(0.079)		(0.076)		(0.077)	
*Dummy_year*	Yes		Yes		Yes		Yes		Yes	
*Constant*	2.329	***	2.841	***	2.803	***	2.633	***	3.026	***
	(0.845)		(0.925)		(0.921)		(0.904)		(0.951)	
*lnalpha_constant*	-0.503	***	-0.365	***	-0.362	***	-0.365	***	-0.373	***
	(0.036)		(0.038)		(0.038)		(0.037)		(0.039)	
*Nb of observations*	19308		19308		19308		19308		19308	
*Nb of groups*	1363		1363		1363		1363		1363	
*R* ^*2*^ *_pseudo*	0.1276		0.1092		0.1092		0.1103		0.1105	
*Loglikelihood*	-74458		-76044		-76027		-75933		-75919	

Note 1:

***, ** and * show significance at the 1%, 5% and 10% levels, respectively, and standard errors are presented in parentheses.

**Table 9:** The regression results of the impact of regional-level network measures on the number of citations.

*Variables*	Models									
*NumCitRegion*_*t*_	(1)		(2)		(3)		(4)		(5)	
*RegionDegCentrality*_*t-1*_	0.229	**								
	(0.055)									
*RegionEigenvector*_*t-1*_			0.005	***						
			(0.010)							
*RegionCloseness*_*t-1*_					0.119	*				
					(0.442)					
*RegionBetweenness*_*t-1*_							0.026	*		
							(0.008)			
*RegionClustering*_*t-1*_									-0.393	
									(0.061)	
*NumInventors*_*t*_	1.557	***	1.705	***	1.7697	***	1.645	***	1.7536	***
	(0.103)		(0.112)		(0.114)		(0.107)		(0.109)	
*PropYearPat*_*t*_	-7.104	***	-8.699	***	-9.294	***	-7.698	***	-8.975	***
	(0.525)		(0.390)		(0.460)		(0.466)		(0.379)	
*ShareWomen*_*t*_	3.447	***	2.961	***	2.821	***	3.051	***	2.908	***
	(0.572)		(0.568)		(0.550)		(0.569)		(0.540)	
*Dummy_year*	Yes		Yes		Yes		Yes		Yes	
*Constant*	-1.301	***	-1.118	***	-0.748	**	-1.416	***	-0.624	**
	(0.256)		(0.287)		(0.300)		(0.269)		(0.261)	
*lnalpha_constant*	0.585	***	0.593	***	0.59	***	0.590	***	0.568	***
	(0.035)		(0.038)		(0.038)		(0.037)		(0.035)	
*Nb of observations*	19308		19308		19308		19308		19308	
*Nb of groups*	1363		1363		1363		1363		1363	
*R* ^*2*^ *_pseudo*	0.0841		0.0823		0.0827		0.0832		0.0857	
*Loglikelihood*	-71522		-71659		-71633		-71594		-71392	

Note 1:

***, ** and * show significance at the 1%, 5% and 10% levels, respectively, and standard errors are presented in parentheses.

When it comes to our regional-level network measures, our analyses provide similar results. The findings suggest that regions that have high number of linkages to other regions within the invention network are more innovative. The closeness, betweenness and eigenvector centralities also show the positive impact on patenting activity and patent citations at the regional level, which indicates that it is important for a region to both be connected to regions that occupy a central position in the co-inventor network and be a broker and connect otherwise disconnected regions. At the same time, the tests of difference in coefficients indicate that the country-level centrality effects are stronger. This may be explained by the fact that even though there are differences in regional innovation structures, international linkages are more driven by country-level dynamics due to the existence of different country level scientific collaboration agreements, as well as country-level policies and inter-country relations. Existing studies in political economy, for instance, demonstrate that regions are more constrained than countries in establishing international collaborative structures within which scientists and firms collaborate [65]. At the same time, existing studies demonstrate that international linkages are most critical for radical innovations [16,35], because they give access to knowledge pools not available locally and regionally. Therefore, country-level centrality in the global network has a stronger impact on innovation, because as demonstrated by our analysis, in the regional network many regions in the developed countries are connected to regions in their respective countries, thus the weight of international linkages in not as pronounced as in the country-level network.

As for the clustering coefficient measure, geographically clustered regions do not seem to produce more patents in our data sample. We used various models in our analysis to identify how clustering influences countries’ and regions’ patenting performance, and our results are significant at the country level only at a 10% confidence interval and not significant at the regional level. Again, country-level network effects seem to be more important in this case too. Clustering coefficient at the country level may be reflecting repeated collaborations between different blocks such as Germany or US led blocks (as shown in the figures above) or BRICs countries (Russia-China collaboration).

Furthermore, we examine the impact of some inventor characteristics on patenting performance. Interestingly, the average number of co-inventors has a positive impact on patenting activity because the number of co-inventors reflects the extent of collaboration between inventors. It seems that patents with a higher number of co-inventors are also more likely to receive more citations.

We also include the average proportion of patents that an inventor from a specific country produces over their active years. The variable *PropYearPat* has a negative impact on the results, which indicates that a longer period between first and last patenting activities is not beneficial when an inventor contributes to few patents.

We also examine whether the fact that patent co-inventors are affiliated with more diverse countries contributes to higher performance. We observe an inverse relationship in a few models. This can be explained by the fact that a specific group of countries (for the most part, the ones that are in the center of our co-inventor networks) seems to be producing high-quality innovations and it is better to develop collaborations strategically with the most innovative countries instead of collaborating with as many countries as possible.

## Discussion of the results related to women inventors

As far as the gender-related aspects of co-inventor networks are concerned, the results in Tables 6 through 9 point to interesting trends. For example, the presence of women inventors in a country’s or region’s co-inventor pool is positively associated with the citation impact of patents. The results suggest that if the share of women inventors among co-inventors is high, the citation impact of patents is improved. However, we could not capture the share of influence on a country’s/region’s total number of patents that is attributable to women inventors. Therefore, we may conclude that the participation of women inventors is more important for the quality of innovations (citations) than their quantity.

We continue with a more comparative analysis by studying the country- and regional-level networks of women inventors. As we mentioned in the methodology section, we constructed these networks by considering only the links between inventors of patents that include at least one woman inventor, and each country’s or region’s position in the country- or regional-level network, respectively, was computed using the patenting activity of women inventors. We use these networks to analyze the impact of country- and regional-level network measures on women inventors’ innovation performance. The results are presented in Tables 10–13. Similar to our previous findings on the impact of centrality measures on patenting activity in the network of all (men and women) inventors, all the centrality measures (except eigenvector centrality) have a significant positive impact on patenting performance in both the country- and regional-level networks of women inventors. One reason for eigenvector centrality’s insignificant results could be the fact that women inventors are not connected to many other women inventors who themselves have high centrality scores. As far as patent citations are concerned, higher centrality measures are indicative of a higher patent citation rate, as we observe in Tables 12 and 13. Degree centrality and closeness centrality have a significant impact on patent citations, whereas betweenness centrality has an insignificant impact, which means that, unlike in the network of all inventors, when women inventors are in an intermediary position, it does not seem to increase patent visibility. Therefore, maintaining cohesive relationships where collaborators are also connected is more important for women-led innovations than the position of power and control associated with brokerage.

**Table 10:** The regression results of the impact of country-level network measures on the number of patents invented by women inventors.

*Variables*	Models									
*NumFemPatCtry*_*t*_	(1)		(2)		(3)		(4)		(5)	
*CtryFemDegCentrality*_*t-1*_	0.323	***								
	(0.089)									
*CtryFemEigenvector*_*t-1*_			0.039							
			(0.041)							
*CtryFemCloseness*_*t-1*_					0.845	***				
					(0.245)					
*CtryFemBetweenness*_*t-1*_							0.016	**		
							(0.007)			
*CtryFemClustering*_*t-1*_									0.106	**
									(0.044)	
*NumInventors*_*t*_	1.133	***	1.120	***	1.109	***	1.120	***	1.176	***
	(0.300)		(0.307)		(0.354)		(0.329)		(0.346)	
*PropYearPat*_*t*_	-1.168	***	-1.246	***	-1.278	***	-1.223	***	-1.271	***
	(0.107)		(0.120)		(0.141)		(0.127)		(0.151)	
*PropWithdrawalPat*_*t*_	-1.511	**	-1.593	**	-0.773		-1.000		-1.263	
	(0.588)		(0.656)		(0.920)		(0.718)		(0.795)	
*ShareWomen*_*t*_	2.112	*	2.222	**	2.221	**	1.971	*	2.040	*
	(1.096)		(1.118)		(1.100)		(1.127)		(1.159)	
*GrossDomesticProduct*_*t*_	0.145	**	0.141	**	0.179	**	0.156	**	0.154	**
	(0.068)		(0.065)		(0.071)		(0.068)		(0.070)	
*Dummy_year*	Yes		Yes		Yes		Yes		Yes	
*Constant*	-4.827	***	-4.499	***	-5.769	***	-4.662	***	-4.511	***
	(1.426)		(1.405)		(1.983)		(1.474)		(1.448)	
*lnalpha_constant*	-1.052	***	-1.026	***	-0.955	***	-1.017	***	-0.990	***
	(0.109)		(0.100)		(0.107)		(0.104)		(0.110)	
*Nb of observations*	349		349		349		349		349	
*Nb of groups*	22		22		22		22		22	
*R* ^*2*^ *_pseudo*	0.1697		0.1673		0.163		0.1664		0.1643	
*Loglikelihood*	-1546		-1550		-1664		-1552		-1556	

Note 1:

***, ** and * show significance at the 1%, 5% and 10% levels, respectively, and standard errors are presented in parentheses.

**Table 11:** The regression results of the impact of country-level network measures on the number of citations of patents invented by women inventors.

*Variables*	Models									
*NumFemCitCtry*_*t*_	(1)		(2)		(3)		(4)		(5)	
*CtryFemDegCentrality*_*t-1*_	0.497	**								
	(0.232)									
*CtryFemEigenvector*_*t-1*_			0.155							
			(0.101)							
*CtryFemCloseness*_*t-1*_					1.466	**				
					(0.705)					
*CtryFemBetweenness*_*t-1*_							0.024			
							(0.016)			
*CtryFemClustering*_*t-1*_									0.261	*
									(0.134)	
*NumInventors*_*t*_	2.818	***	2.886	***	2.798	***	2.792	***	2.936	***
	(0.808)		(0.811)		(0.879)		(0.871)		(0.912)	
*PropWithdrawalPat*_*t*_	-3.389	*	-3.681	**	-1.276		-2.184		-2.855	*
	(1.734)		(1.641)		(1.555)		(1.636)		(1.628)	
*ShareWomen*_*t*_	5.981	***	6.424	***	6.313	***	5.745	**	5.762	**
	(2.123)		(1.995)		(2.296)		(2.246)		(2.251)	
*GrossDomesticProduct*_*t*_	0.518	***	0.545	***	0.618	***	0.583	***	0.599	***
	(0.126)		(0.141)		(0.162)		(0.143)		(0.163)	
*Dummy_year*	Yes		Yes		Yes		Yes		Yes	
*Constant*	-15.32	***	-12.25	***	-16.11	***	-12.845	***	-13.062	***
	(2.403)		(2.812)		(3.753)		(2.761)		(3.249)	
*lnalpha_constant*	0.147		0.168		0.214		0.183		0.190	
	(0.165)		(0.169)		(0.152)		(0.159)		(0.160)	
*Nb of observations*	349		349		349		349		349	
*Nb of groups*	22		22		22		22		22	
*R* ^*2*^ *_pseudo*	0.0906		0.0886		0.0849		0.087		0.0866	
*Loglikelihood*	-1687		-1690		-1811		-1693		-1694	

Note 1:

***, ** and * show significance at the 1%, 5% and 10% levels, respectively, and standard errors are presented in parentheses.

**Table 12:** The regression results of the impact of regional-level network measures on the number of patents invented by women inventors.

*Variables*	Models									
*NumFemPatRegion*_*t*_	(1)		(2)		(3)		(4)		(5)	
*RegionFemDegCentrality*_*t-1*_	0.189	***								
	(0.062)									
*RegionFemEigenvector*_*t-1*_			0.0239	*						
			(0.011)							
*RegionFemCloseness*_*t-1*_					0.384	***				
					0.2523					
*RegionFemBetweenness*_*t-1*_							0.013	*		
							(0.007)			
*RegionFemClustering*_*t-1*_									0.0166	**
									(0.014)	
*NumInventors*_*t*_	0.742	***	0.762	***	0.7578	***	0.703	***	0.7694	***
	(0.162)		(0.113)		(0.129)		(0.112)		(0.127)	
*PropYearPat*_*t*_	-14.741	***	-15.1973	***	-15.963	***	-15.292	***	-15.9313	***
	(1.117)		(0.948)		0.9863		(1.168)		(1.033)	
*PropWithdrawalPat*_*t*_	-1.454	**	-1.1466	*	-1.2251	*	-1.364	**	-1.3483	**
	(0.670)		(0.620)		(0.634)		(0.679)		(0.639)	
*ShareWomen*_*t*_	0.784	*	0.0116	**	0.478	*	0.050	*	0.0872	*
	(0.856)		(0.526)		(0.495)		(0.580)		(0.541)	
*Dummy_year*	Yes		Yes		Yes		Yes		Yes	
*Constant*	0.432	***	0.0542		0.6076	*	0.616	*	0.7151	**
	(0.337)		(0.430)		(0.312)		(0.316)		(0.306)	
*lnalpha_constant*	-0.385	***	-0.3588	***	-0.3676	***	-0.370	***	-0.3662	***
	(0.071)		(0.068)		(0.073)		(0.072)		(0.073)	
*Nb of observations*	6768		6768		6768		6768		6768	
*Nb of groups*	513		513		513		513		513	
*R* ^*2*^ *_pseudo*	0.0805		0.0788		0.0776		0.0784		0.0774	
*Loglikelihood*	-32114		-34811		-32217		-32187		-32222	

Note 1:

***, ** and * show significance at the 1%, 5% and 10% levels, respectively, and standard errors are presented in parentheses.

**Table 13:** The regression results of the impact of regional-level network measures on the number of citations of patents invented by women inventors.

*Variables*	Models									
*NumFemCitRegion*_*t*_	(1)		(2)		(3)		(4)		(5)	
*RegionFemDegCentrality*_*t-1*_	0.182	**								
	(0.084)									
*RegionFemEigenvector*_*t-1*_			0.0301	*						
			(0.018)							
*RegionFemCloseness*_*t-1*_					1.3324	***				
					(0.321)					
*RegionFemBetweenness*_*t-1*_							0.012			
							(0.009)			
*RegionFemClustering*_*t-1*_									0.0108	*
									(0.024)	
*NumInventors*_*t*_	1.477	***	1.6819	***	1.6173	***	1.617	***	1.7022	***
	(0.163)		(0.163)		(0.196)		(0.162)		(0.185)	
*PropWithdrawalPat*_*t*_	-0.341		-0.2546		-0.0949		-0.172		-0.126	
	(0.545)		(0.737)		(0.738)		(0.782)		(0.777)	
*ShareWomen*_*t*_	2.036	***	2.1352	***	1.875	***	2.135	***	2.1924	***
	(0.679)		(0.629)		(0.586)		(0.665)		(0.643)	
*Dummy_year*	Yes		Yes		Yes		Yes		Yes	
*Constant*	-1.881	***	-2.7053	***	-2.2387	***	-1.922	***	-1.9309	***
	(0.374)		(0.581)		(0.399)		(0.388)		(0.390)	
*lnalpha_constant*	0.313	***	0.3457	***	0.3049	***	0.322	***	0.3254	***
	(0.055)		(0.054)		0.059		(0.057)		(0.059)	
*Nb of observations*	6768		6768		6768		6768		6768	
*Nb of groups*	513		513		513		513		513	
*R* ^*2*^ *_pseudo*	0.0713		0.0707		0.0721		0.0702		0.0698	
*Loglikelihood*	-32152		-34660		-32125		-32189		-32204	

Note 1:

***, ** and * show significance at the 1%, 5% and 10% levels, respectively, and standard errors are presented in parentheses.

When we analyze the clustering coefficient of countries and regions the results show that, unlike in the network of all inventors, clustering between countries and regions in the network of women inventors has a significant positive effect on the number of patents at the country and regional levels, respectively, in addition to increasing the citation impact of patents.

Therefore, we can accept Propositions 1a and 1b that network centrality positively affects a country’s/region’s innovation performance. These results are generally consistent with the studies of co-inventor networks at an individual level that are mentioned in the theoretical part of the paper (42; 44; 43; 58) and contribute to the emerging literature on inter-regional linkages and their effects on innovation [41]. They also make an original contribution to the literature on innovation analysis at the country level, which has so far focused on indicators such as FDI flow, trade, social capital and economic complexity linkages [5–7,37).

As far as the gender-related aspects of co-inventor networks are concerned, the results of the study suggest that centrality measures are beneficial in fostering innovation activity in both the country- and regional-level networks of women inventors, which supports our Propositions 4a and 4b, respectively. Our analysis indicates that the share of women inventors in the pool of inventors does not have an impact on the number of patents produced by the country or the region. At the same time, we find a significant positive association with the number of patent citations, which indicates that the participation of women inventors is crucial for the quality of innovation rather than the quantity. Therefore, our Propositions 3a and 3b are only partially supported. These findings make an original contribution to both country and regional studies of innovation [36,40], as well as gender literature that so far focused more on the examination of gender diversity in R&D teams and organisations [34,35].

As far as clustering is concerned, while some studies indicate that being in a cluster can be fruitful, we found that clustering is only marginally significant at the country level, and is not significant at the regional level. At the same time, we find clustering has a significant impact in the network of women inventors at both the county and regional levels. Therefore, our Propositions 2a and 2b regarding the country and regional networks of all inventors, respectively, are partially supported; and our Propositions 5a and 5b regarding the country and regional networks of women inventors, respectively, are supported. While there is no country- or regional-level innovation literature that helps explain this phenomenon, insights from the literature on gender shed light on it. Gender studies indicate that women are more likely to succeed in collaborative groups in which their collaborators are also linked because women’s productivity is positively influenced by the levels of trust and social capital that exist, which are usually high in cohesive groups [60] and women are also more prone to repeat their existing collaborations for this reason [61,62]. Therefore, countries and regions that are situated in a cohesive cluster in the network of women inventors see their innovation performance positively affected.

As a robustness, check, we conducted an analysis comparing inventors with at least 5 patents to those with at least 10 patents and found that the results related to the importance of the network centrality indicators were highly consistent, demonstrating the robustness of our conclusions. Notable differences were found in the impact of clustering on patent output. Clustered linkages (dense repeated collaborations) between prolific women inventors had a stronger impact on country and regional innovation than clustered linkages between women inventors who published five patents. Additionally, the positive impact of the ShareWomen variable was more consistent and robust in the 10-patent analysis compared to the 5-patent analysis. These results suggest that dense interactions, as well as a higher proportion of female inventors in countries and regions has a stronger impact on the innovation of prolific inventors. Therefore, these findings indicate that for consistent innovation network embeddedness and gender diversity play a crucial role.

The results of the robustness analysis are provided in appendix D in S1 Appendix of the paper.

## Conclusions and policy implications

The purpose of this study is to investigate the relationship between collaboration and innovation performance in various countries and regions as assessed through their patenting activity over the period from 1978 to 2019 with a particular focus on women inventors. According to the existing literature, patents are a key driver of innovation and provide a valuable measure of countries’/regions’ inventiveness [15] because innovations emerge from the complex collaborations and interactions that exist among a diverse set of inventors. Various studies have proven, using theoretical and empirical approaches, that there is a relationship between collaboration and innovation performance [17]. In this paper, we intended to assess the relationship between collaboration and innovation performance at the country and regional levels with a focus on women inventors by applying a social network analysis approach. Using the social network perspective on collaboration helped us to focus on the structure of the relationships between the countries or regions collaborating in a co-invention network. The results of our econometric analysis reveal that centrality in the co-inventor network has a positive effect on patenting performance at both the country and regional levels. We conclude that more innovative countries and regions are more likely to have a higher number of innovative ties with other countries and regions. However, these results are also nuanced by the insight that it is best to have a strategic approach to collaborations and it is more important to develop connections with more central and innovative countries/regions than as many countries/regions as possible as is evidenced by the importance of the closeness and eigenvector centralities. We find similar results at the regional level.

At the same time, our analysis demonstrates important nuances in the differences between the country-level and regional level dynamics, because country-level network centrality effects have a stronger impact on innovation that the regional-level ones, while clustering coefficient is even significant only at the country level. These results have important implications for the federal level innovation policies and demonstrate the importance of inter-country collaborative agreements that give an umbrella to scientists to collaborate.

As far as the gender-related aspect is concerned, the results show that the patenting activity of women inventors grew over time, as evidenced by an increasing number of patents and additional collaboration activity between countries. The density of the network of women inventors increased over the period studied as more countries became visible in the network, which indicates that there was an important evolution among women inventors around the globe. At the same time, women inventors were most prolific in Germany and the US. At the regional level, we found that the regions that are home to big global cities account for a considerable share of the geographical distribution of women inventors.

As far as the network aspects are concerned, we find that the average share of women inventors in the pool of inventors working on a patent is positively correlated with the quality of countries’/regions’ innovations as revealed by a positive effect on patent citations. Therefore, gender diversity is important for innovation quality. And our robustness analysis nuances that gender diversity is particularly important for innovations conducted by prolific inventors. A diverse group of inventors can draw on different sources of knowledge and different perspectives, which is critical for consistent innovation. The literature on gender indicates that men and women innovate differently [32,56,57]; therefore, countries and regions that encourage and support gender diversity in inventor collaborations will see an increase on the quality of their innovations: they will have a broader scope of innovations and their innovations will be more impactful. Our study also indicates that in the case of the women inventor network, cohesive groups of countries and regions (that have many linkages among their women inventors) see their innovation performance get an additional boost, particularly, in the case of their prolific women inventors whose innovation turned out to be especially dependent on dense and repeated collaborations.

Our findings yield important implications for policymakers. Countries and regions that have more people working in science and technology are more likely to successfully develop innovations; however, monetary investment in innovation is not sufficient as our results show that the most innovative countries and regions are those that are central in a network of inventors and those that have higher gender diversity in their innovation teams. These results indicate that the development of countries’/regions’ innovation processes requires deliberate approaches and policies that focus on the connections between inventors. Countries and regions need to invest in the development of collaborations between their inventors and inventors in the countries and regions that are the most productive in terms of innovation. Moreover, our analysis points to the stronger effect of the country-level network measures indicating that federal innovation policies and collaboration agreements with other countries play an important role with providing a framework for the establishment and maintenance of international linkages between inventors, which has an important impact on innovation.

Additionally, enhancing interactions between men and women inventors in their respective innovation systems should be prioritized. Our findings indicate that innovation among women inventors is particularly influenced by group cohesion, so involving women inventors in repeated collaborations with inventors from inter-connected regions and countries will help increase their innovation performance. Inventors are key drivers of a country’s/region’s innovation system, and countries and regions need to introduce policies beyond core innovation policies to improve research quality and innovation investment for women inventors. Women inventors have high innovation potential, and public policies can help them by supporting innovation productivity growth and making the flow of knowledge between men and women inventors smoother.

Policymakers in different countries and regions have started to realize the importance of collaborations and have developed policies aiming at creating collaborative platforms and structures, such as the supercluster policy by the Canadian government that aims at strengthening collaborations between different regions, as well as between Canadian innovation system and other countries, or the Horizon Research and Development program by the European Union where applicants are specifically encouraged to include international partners. Given our findings, such efforts may be more targeted based on the analysis of inventor network properties and also their gender-related aspects: for instance, they can prioritize collaborations with highly connected countries and regions (with a particular focus on international connections), as well as encourage gender diversity in inventor teams.

When it comes to future research, this study could be built upon by evaluating and comparing patents in different disciplines and investigating how collaboration effectiveness impacts women inventors’ innovation productivity in different industries. We believe that this is a critical matter for the development of countries’/regions’ innovation systems.

One of the limitations of this study is our data sampling. The data for the networks was collected from OECD database that relies on PATSTAT maintained by EPO (European Patent Office). While PATSTAT contains impressive data relating to more than 100 million patent documents from leading industrialised and developing countries, it may still not have information on some patents in developing countries that were filed with the national patent offices. Another limitation of our study is that we mostly focus on prolific inventors (those who have at least ten patents over the period considered) and inventors who published five patents in our empirical analysis. We chose to focus on such inventors due to several reasons discussed in the empirical section of the paper. Future studies could explore if our results hold with the network of all the inventors, even including inventors who published just one patent. This study also has a limitation in terms of gender, because gender should be self-reported. We used inventors’ names to determine their gender rather than asking them what their gender was. Moreover, the results of our study may be affected by inventor mobility, as we, unfortunately, did not have access to their mobility data.

## Supporting information

S1 Fig (PNG)

S2 Fig (PNG)

S1 Data (ZIP)

S1 Appendix (DOCX)
