# Supplementary figures and images for: Economic geography of innovation: The effect of gender-related aspects of co-inventor networks on country and regional innovation
Source: PLoS One. 2023 Jul 27;18(7):e0288843. doi: 10.1371/journal.pone.0288843 (PMC10374134; doi:10.1371/journal.pone.0288843)

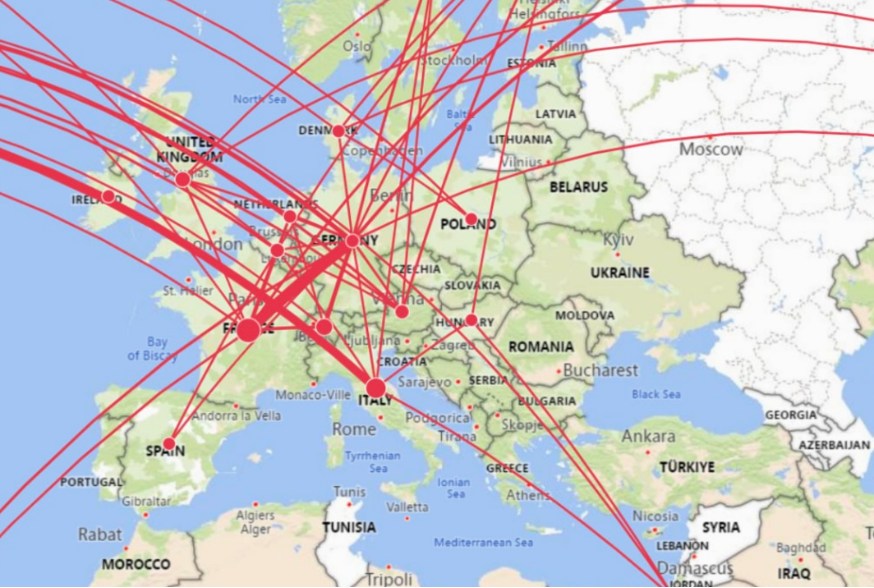

Supplement: S1 Fig — (PNG) [file pone.0288843.s001.png]

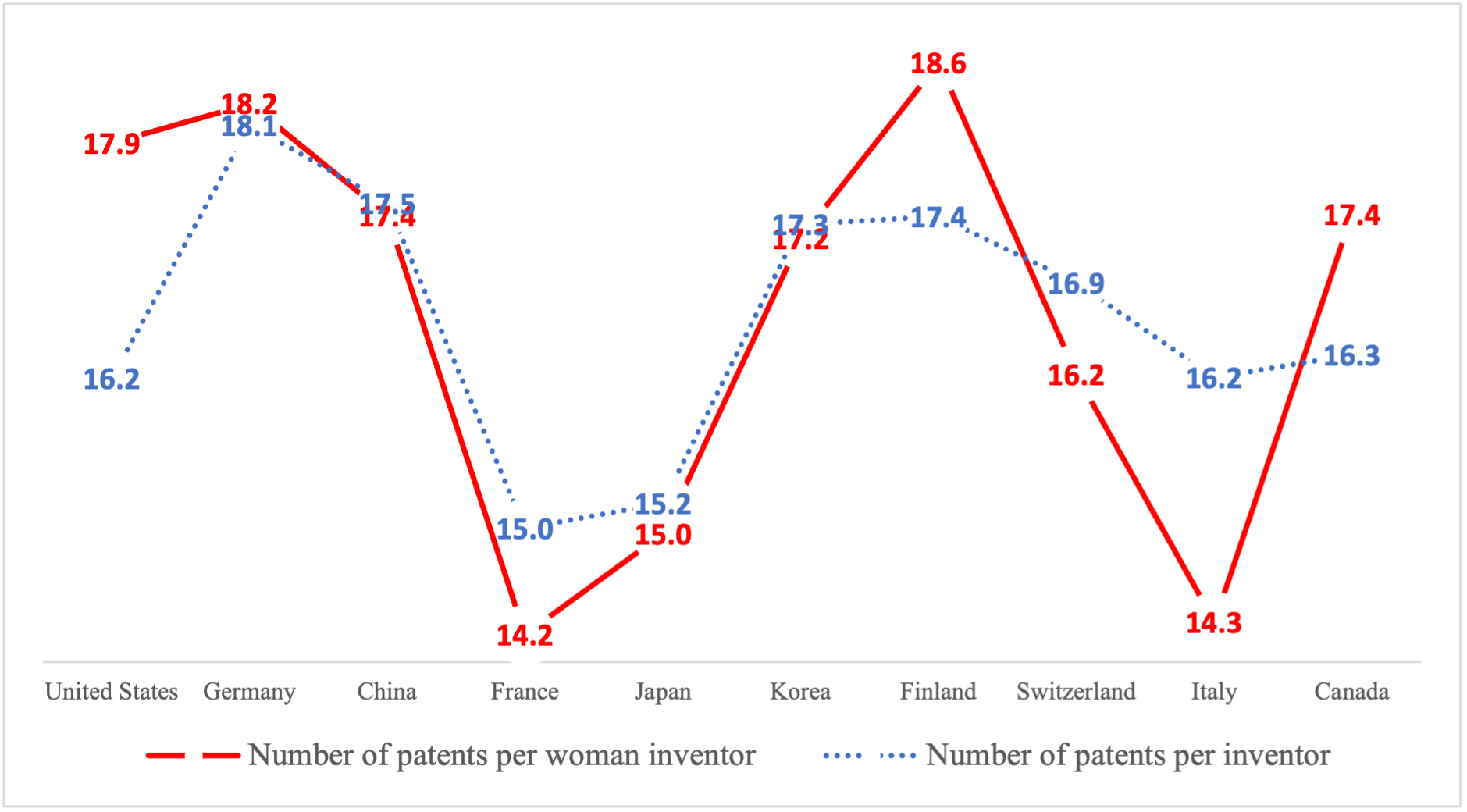

Supplement: S2 Fig — (PNG) [file pone.0288843.s002.png]
