# Supplementary material for: Economic geography of innovation: The effect of gender-related aspects of co-inventor networks on country and regional innovation
Source: PLoS One. 2023 Jul 27;18(7):e0288843. doi: 10.1371/journal.pone.0288843 (PMC10374134; doi:10.1371/journal.pone.0288843)
Supplement: S1 Appendix — (DOCX) [file pone.0288843.s004.docx]

*Appendices*

| ***Variables*** (***Country level)*** | ***Obs*** | ***Mean*** | ***Std. Dev.*** | ***Min*** | ***Max*** |
| --- | --- | --- | --- | --- | --- |
| *NumPatCtry_t_* | 771 | 823.329 | 2193.730 | 1.000 | 14552 |
| *NumCitCtry_t_* | 771 | 945.241 | 2859.308 | 0.000 | 27769 |
| *CtryDegCentrality_t_* | 771 | 4.459 | 5.033 | 0.000 | 25 |
| *CtryEigenvector_t_* | 771 | 228.292 | 181.799 | 3.000 | 540 |
| *CtryCloseness_t_* | 771 | 0.448 | 0.243 | 0.000 | 1 |
| *CtryBetweenness_t_* | 771 | 0.026 | 0.066 | 0.000 | 0.487 |
| *CtryClustering_t_* | 771 | 0.403 | 0.386 | 0.000 | 1 |
| *NumInventors_t_* | 771 | 3.737 | 1.423 | 1.000 | 14.804 |
| *PropYearPat_t_* | 771 | 0.027 | 0.043 | 0.000 | 0.239 |
| *PropWithdrawalPat_t_* | 771 | 0.269 | 0.072 | 0.106 | 0.443 |
| *ShareWomen_t_* | 771 | 0.095 | 0.083 | 0.000 | 0.714 |
| *ShareCountry_t_* | 771 | 1.256 | 0.330 | 1.000 | 4.000 |
| *GrossDomesticProduct_t_* | 771 | 1,983,836 | 3,567,730 | 15,769 | 23,000,000 |
| *ShareInventor_t_* | 771 | 0.421 | 0.137 | 0.088 | 1.000 |
|  |  |  |  |  |  |

Appendix A1 – Descriptive Statistics – All Inventors

Appendix A2 – Descriptive Statistics – Women Inventors

| ***Variables (Country level)*** | ***Obs*** | ***Mean*** | ***Std. Dev.*** | ***Min*** | ***Max*** |
| --- | --- | --- | --- | --- | --- |
| *NumFemPatCtry_t_* | 349 | 79.626 | 162.399 | 1.000 | 863.000 |
| *NumFemCitCtry_t_* | 349 | 99.603 | 207.788 | 0.000 | 1359.000 |
| *CtryFemDegCentrality_t_* | 349 | 2.143 | 2.448 | 0.000 | 11.000 |
| *CtryFemEigenvector_t_* | 349 | 180.190 | 86.920 | 2.000 | 262.000 |
| *CtryFemCloseness_t_* | 349 | 0.349 | 0.276 | 0.000 | 1.000 |
| *CtryFemBetweenness_t_* | 349 | 0.021 | 0.046 | 0.000 | 0.272 |
| *CtryFemClustering_t_* | 349 | 0.284 | 0.377 | 0.000 | 1.000 |
| *NumInventors_t_* | 349 | 4.338 | 1.254 | 1.000 | 12.000 |
| *PropYearPat_t_* | 349 | 0.058 | 0.107 | 0.000 | 1.600 |
| *PropWithdrawalPat_t_* | 349 | 0.325 | 0.102 | 0.000 | 0.602 |
| *ShareWomen_t_* | 349 | 0.462 | 0.119 | 0.125 | 1.000 |
| *ShareCountry_t_* | 349 | 1.262 | 0.315 | 1.000 | 2.714 |
| *GrossDomesticProduct_t_* | 349 | 1,881,865 | 2,757,391 | 56,140 | 21,700,000 |
| *ShareInventor_t_* | 349 | 0.327 | 0.109 | 0.083 | 1.000 |
|  |  |  |  |  |  |

| ***Variables (Region level)*** | ***Obs*** | ***Mean*** | ***Std. Dev.*** | ***Min*** | ***Max*** |
| --- | --- | --- | --- | --- | --- |
| *NumFemPatRegion_t_* | 6768 | 30.92 | 85.49 | 1.00 | 2872.00 |
| *NumFemCitregion_t_* | 6768 | 35.76 | 147.48 | 0.00 | 10264.00 |
| *RegionFemDegCentrality_t_* | 6768 | 16.05 | 18.85 | 0.00 | 119.00 |
| *RegionFemEigenvector_t_* | 6768 | 0.13 | 0.20 | 0.00 | 1.00 |
| *RegionFemCloseness_t_* | 6768 | 0.30 | 0.12 | 0.00 | 1.00 |
| *RegionFemBetweenness_t_* | 6768 | 0.00 | 0.01 | 0.00 | 0.06 |
| *RegionFemClustering_t_* | 6768 | 0.33 | 0.27 | 0.00 | 1.00 |
| *NumInventors_t_* | 6768 | 3.59 | 1.72 | 1.00 | 33.67 |
| *PropYearPat_t_* | 6768 | 0.14 | 0.17 | 0.00 | 3.17 |
| *PropWithdrawalPat_t_* | 6768 | 0.27 | 0.12 | 0.00 | 1.00 |
| *ShareWomen_t_* | 6768 | 0.07 | 0.10 | 0.00 | 1.00 |
| *ShareCountry_t_* | 6768 | 1.14 | 0.28 | 1.00 | 6.00 |
| *ShareInventor_t_* | 6768 | 0.44 | 0.21 | 0.04 | 1.00 |
|  |  |  |  |  |  |

Appendix B

Table B1 – Percentage of patents produced by gender and IPC code category

| **IPC Code Categories** | **Percentage of Patents Produced by Women** | **Percentage of Patents Produced by Men** |
| --- | --- | --- |
| CHEMISTRY; METALLURGY | 9% | 18% |
| ELECTRICITY | 4% | 10% |
| FIXED CONSTRUCTIONS | 0% | 3% |
| HUMAN NECESSITIES | 4% | 8% |
| MECHANICAL ENGINEERING; LIGHTING; HEATING; WEAPONS; BLASTING | 2% | 9% |
| PERFORMING OPERATIONS; TRANSPORTING | 3% | 16% |
| PHYSICS | 3% | 8% |
| TEXTILES; PAPER | 1% | 3% |
| **Total** | **25%** | **75%** |

Appendix C

Figure C1 – A closer look at the women inventor network at the country level

Figure C2 – The number of patents per inventor and per woman inventor in the 10 countries having the highest number of women inventors

Appendix D

Table D-1 – The regression results of the impact of country-level network measures on the number of patents_5 patents

| ***Variables*** | **Models** | | | | | | | | | |
| --- | --- | --- | --- | --- | --- | --- | --- | --- | --- | --- |
| ***NumPatCtry_t_*** | **(1)** | | **(2)** | | **(3)** | | **(4)** | | **(5)** | |
| *CtryDegCentrality_t-1_* | 1.174 | *** |  |  |  |  |  |  |  |  |
|  | (0.135) |  |  |  |  |  |  |  |  |  |
| *CtryEigenvector_t-1_* |  |  | 0.268 | *** |  |  |  |  |  |  |
|  |  |  | (0.067) |  |  |  |  |  |  |  |
| *CtryCloseness_t-1_* |  |  |  |  | 4.959 | *** |  |  |  |  |
|  |  |  |  |  | (0.535) |  |  |  |  |  |
| *CtryBetweenness_t-1_* |  |  |  |  |  |  | 0.089 | *** |  |  |
|  |  |  |  |  |  |  | (0.016) |  |  |  |
| *CtryClustering_t-1_* |  |  |  |  |  |  |  |  | 0.064 |  |
|  |  |  |  |  |  |  |  |  | (0.187) |  |
| *NumInventors_t_* | 0.583 |  | 0.367 |  | 0.436 |  | 0.726 |  | 0.425 |  |
|  | (0.440) |  | (0.426) |  | (0.359) |  | (0.553) |  | (0.591) |  |
| *PropYearPat_t_* | -9.823 | *** | -13.807 | *** | -13.707 | *** | -12.183 | *** | -16.300 | *** |
|  | (2.013) |  | (1.803) |  | (2.109) |  | (2.051) |  | (2.521) |  |
| *ShareWomen_t_* | -1.763 |  | -1.566 |  | -2.558 |  | -3.752 |  | -5.291 |  |
|  | (1.259) |  | (1.409) |  | (1.536) |  | (1.417) |  | (1.897) |  |
| *GrossDomesticProduct_t_* | 0.531 | *** | 0.499 | *** | 0.615 | *** | 0.642 | *** | 0.829 | *** |
|  | (0.083) |  | (0.097) |  | (0.083) |  | (0.098) |  | (0.113) |  |
| *Dummy_year* | Yes |  | Yes |  | Yes |  | Yes |  | Yes |  |
| *Constant* | -3.351 | *** | -2.427 | * | -4.330 | *** | -4.518 | *** | -5.804 | *** |
|  | (1.107) |  | (1.343) |  | (1.109) |  | (1.186) |  | (1.414) |  |
| *lnalpha_constant* | -0.195 |  | -0.158 |  | -0.004 |  | -0.017 |  | 0.225 |  |
|  | (0.130) |  | (0.122) |  | (0.138) |  | (0.118) |  | (0.141) |  |
| *Nb of observations* | 991 |  | 991 |  | 991 |  | 991 |  | 991 |  |
| *Nb of groups* | 40 |  | 40 |  | 40 |  | 40 |  | 40 |  |
| *R^2^_pseudo* | 0.1249 |  | 0.122 |  | 0.1093 |  | 0.1111 |  | 0.0908 |  |
| *Loglikelihood* | -6528 |  | -6549 |  | -6644 |  | -6631 |  | -6782 |  |

Note 1: ***, ** and * show significance at the 1%, 5% and 10% levels, respectively, and standard errors are presented in parentheses.

Table D-2 – The regression results of the impact of country-level network measures on the number of citations_5patants

| ***Variables*** | **Models** | | | | | | | | | | | | | |
| --- | --- | --- | --- | --- | --- | --- | --- | --- | --- | --- | --- | --- | --- | --- |
| ***NumCitCtry_t_*** | **(1)** | | **(2)** | | | **(3)** | | | **(4)** | | | **(5)** | | |
| *CtryDegCentrality_t-1_* | 0.930 | *** | |  |  | |  |  | |  |  | |  |  |
|  | (0.156) |  | |  |  | |  |  | |  |  | |  |  |
| *CtryEigenvector_t-1_* |  |  | | 0.065 | *** | |  |  | |  |  | |  |  |
|  |  |  | | (0.013) |  | |  |  | |  |  | |  |  |
| *CtryCloseness_t-1_* |  |  | |  |  | | 3.992 | *** | |  |  | |  |  |
|  |  |  | |  |  | | (0.555) |  | |  |  | |  |  |
| *CtryBetweenness_t-1_* |  |  | |  |  | |  |  | | 0.071 | *** | |  |  |
|  |  |  | |  |  | |  |  | | (0.016) |  | |  |  |
| *CtryClustering_t-1_* |  |  | |  |  | |  |  | |  |  | | -0.218 |  |
|  |  |  | |  |  | |  |  | |  |  | | (0.165) |  |
| *NumInventors_t_* | -13.323 |  | | -17.281 |  | | -16.525 |  | | -14.981 |  | | -20.207 |  |
|  | (2.700) |  | | (2.856) |  | | (2.624) |  | | (2.701) |  | | (2.750) |  |
| *PropYearPat_t_* | -1.376 |  | | -3.398 |  | | -2.079 |  | | -2.904 |  | | -4.270 | ** |
|  | (1.646) |  | | (2.084) |  | | (1.750) |  | | (1.780) |  | | (2.061) |  |
| *ShareWomen_t_* | -0.204 |  | | -0.128 |  | | -0.081 |  | | -0.252 |  | | -0.207 |  |
|  | (0.440) |  | | (0.468) |  | | (0.399) |  | | (0.495) |  | | (0.566) |  |
| *GrossDomesticProduct_t_* | 0.544 | *** | | 0.745 | *** | | 0.622 | *** | | 0.649 | *** | | 0.802 | *** |
|  | (0.108) |  | | (0.120) |  | | (0.104) |  | | (0.113) |  | | (0.115) |  |
| *Dummy_year* | Yes |  | | Yes |  | | Yes |  | | Yes |  | | Yes |  |
| *Constant* | -4.734 | *** | | -7.243 | *** | | -6.078 | *** | | -5.662 | *** | | -6.884 | *** |
|  | (1.685) |  | | (1.844) |  | | (1.638) |  | | (1.879) |  | | (1.981) |  |
| *lnalpha_constant* | 0.423 | *** | | 0.525 | *** | | 0.463 | *** | | 0.455 | *** | | 0.557 | *** |
|  | (0.112) |  | | (0.126) |  | | (0.121) |  | | (0.113) |  | | (0.132) |  |
| *Nb of observations* | 991 |  | | 991 |  | | 991 |  | | 991 |  | | 991 |  |
| *Nb of groups* | 40 |  | | 40 |  | | 40 |  | | 40 |  | | 40 |  |
| *R^2^_pseudo* | 0.0981 |  | | 0.0897 |  | | 0.0947 |  | | 0.0956 |  | | 0.0873 |  |
| *Loglikelihood* | -6348 |  | | -6408 |  | | -6373 |  | | -6366 |  | | -6424 |  |

Note 1: ***, ** and * show significance at the 1%, 5% and 10% levels, respectively, and standard errors are presented in parentheses.

Table D-3 – The regression results of the impact of country-level network measures on the number of patents invented by women inventors_5 patents

| ***Variables*** | **Models** | | | | | | | | | |
| --- | --- | --- | --- | --- | --- | --- | --- | --- | --- | --- |
| ***NumFemPatCtry_t_*** | **(1)** |  | **(2)** |  | **(3)** |  | **(4)** |  | **(5)** |  |
| *CtryFemDegCentrality_t-1_* | 0.393 | *** |  |  |  |  |  |  |  |  |
|  | (0.085) |  |  |  |  |  |  |  |  |  |
| *CtryFemEigenvector_t-1_* |  |  | 0.024 | ** |  |  |  |  |  |  |
|  |  |  | (0.010) |  |  |  |  |  |  |  |
| *CtryFemCloseness_t-1_* |  |  |  |  | 0.843 | *** |  |  |  |  |
|  |  |  |  |  | (0.323) |  |  |  |  |  |
| *CtryFemBetweenness_t-1_* |  |  |  |  |  |  | 0.017 | ** |  |  |
|  |  |  |  |  |  |  | (0.007) |  |  |  |
| *CtryFemClustering_t-1_* |  |  |  |  |  |  |  |  | -0.110 |  |
|  |  |  |  |  |  |  |  |  | (0.039) |  |
| *NumInventors_t_* | 1.269 | *** | 1.293 | *** | 1.284 | *** | 1.224 | *** | 1.336 | *** |
|  | (0.235) |  | (0.240) |  | (0.244) |  | (0.232) |  | (0.239) |  |
| *PropYearPat_t_* | -1.260 | *** | -1.419 | *** | -1.385 | *** | -1.344 | *** | -1.446 | *** |
|  | (0.090) |  | (0.109) |  | (0.102) |  | (0.111) |  | (0.104) |  |
| *PropWithdrawalPat_t_* | 1.543 | * | 2.643 | ** | 1.978 | * | 2.450 | ** | 2.617 | ** |
|  | (0.805) |  | (1.091) |  | (1.042) |  | (0.980) |  | (1.068) |  |
| *ShareWomen_t_* | 1.540 | * | 1.508 |  | 1.584 | * | 1.727 |  | 1.844 | * |
|  | (0.907) |  | (0.942) |  | (0.962) |  | (0.945) |  | (0.978) |  |
| *GrossDomesticProduct_t_* | 0.268 | *** | 0.279 | *** | 0.293 | *** | 0.305 | *** | 0.271 | *** |
|  | (0.051) |  | (0.061) |  | (0.057) |  | (0.058) |  | (0.064) |  |
| *Dummy_year* | Yes |  | Yes |  | Yes |  | Yes |  | Yes |  |
| *Constant* | -6.134 | *** | -5.992 | *** | -6.051 | *** | -6.277 | *** | -5.630 | *** |
|  | (1.584) |  | (1.773) |  | (1.720) |  | (1.734) |  | (1.791) |  |
| *lnalpha_constant* | -1.032 | *** | -0.951 | *** | -0.956 | *** | -0.940 | *** | -0.944 | *** |
|  | (0.172) |  | (0.154) |  | (0.157) |  | (0.160) |  | (0.154) |  |
| *Nb of observations* | 596 |  | 576 |  | 596 |  | 596 |  | 596 |  |
| *Nb of groups* | 30 |  | 30 |  | 30 |  | 30 |  | 30 |  |
| *R^2^_pseudo* | 0.1959 |  | 0.1882 |  | 0.1894 |  | 0.1884 |  | 0.1886 |  |
| *Loglikelihood* | -2768 |  | -2713 |  | -2790 |  | -2794 |  | -2793 |  |

Note 1: ***, ** and * show significance at the 1%, 5% and 10% levels, respectively, and standard errors are presented in parentheses.

Table D-4 – The regression results of the impact of country-level network measures on the number of citations of patents invented by women inventors _5 patents

| ***Variables*** | | **Models** | | | | | | | | | | | | | | | | | | |  |  |
| --- | --- | --- | --- | --- | --- | --- | --- | --- | --- | --- | --- | --- | --- | --- | --- | --- | --- | --- | --- | --- | --- | --- |
| ***NumFemCitCtry_t_*** | **(1)** | |  | | **(2)** | |  | | **(3)** | |  | | **(4)** | |  | | **(5)** | |  | | | |
| *CtryFemDegCentrality_t-1_* | 0.622 | | | *** | |  | |  | |  | |  | |  | |  | |  | |  | | |
|  | (0.200) | | |  | |  | |  | |  | |  | |  | |  | |  | |  | | |
| *CtryFemEigenvector_t-1_* |  | | |  | | 0.038 | | *** | |  | |  | |  | |  | |  | |  | | |
|  |  | | |  | | (0.014) | |  | |  | |  | |  | |  | |  | |  | | |
| *CtryFemCloseness_t-1_* |  | | |  | |  | |  | | 1.704 | | *** | |  | |  | |  | |  | | |
|  |  | | |  | |  | |  | | (0.495) | |  | |  | |  | |  | |  | | |
| *CtryFemBetweenness_t-1_* |  | | |  | |  | |  | |  | |  | | 0.043 | | *** | |  | |  | | |
|  |  | | |  | |  | |  | |  | |  | | (0.014) | |  | |  | |  | | |
| *CtryFemClustering_t-1_* |  | | |  | |  | |  | |  | |  | |  | |  | | -0.117 | |  | | |
|  |  | | |  | |  | |  | |  | |  | |  | |  | | (0.054) | |  | | |
| *NumInventors_t_* | 1.748 | | |  | | 1.861 | |  | | 1.753 | |  | | 1.575 | |  | | 1.782 | |  | | |
|  | (1.087) | | |  | | (1.225) | |  | | (1.170) | |  | | (1.088) | |  | | (1.183) | |  | | |
| *PropWithdrawalPat_t_* | 0.059 | | |  | | 1.598 | |  | | 0.631 | |  | | 1.486 | |  | | 1.861 | |  | | |
|  | (2.043) | | |  | | (1.842) | |  | | (1.792) | |  | | (1.702) | |  | | (1.705) | |  | | |
| *ShareWomen_t_* | 4.051 | | | *** | | 4.419 | | ** | | 4.384 | | *** | | 4.537 | | *** | | 4.919 | | *** | | |
|  | (1.557) | | |  | | (1.782) | |  | | (1.691) | |  | | (1.624) | |  | | (1.823) | |  | | |
| *GrossDomesticProduct_t_* | 0.715 | | | *** | | 0.841 | | *** | | 0.831 | | *** | | 0.809 | | *** | | 0.872 | | *** | | |
|  | (0.114) | | |  | | (0.116) | |  | | (0.107) | |  | | (0.104) | |  | | (0.115) | |  | | |
| *Dummy_year* | Yes | | |  | | Yes | |  | | Yes | |  | | Yes | |  | | Yes | |  | | |
| *Constant* | -16.432 | | | *** | | -15.961 | | *** | | -15.363 | | *** | | -15.073 | | *** | | -16.211 | | *** | | |
|  | (2.313) | | |  | | (2.076) | |  | | (2.007) | |  | | (1.941) | |  | | (2.081) | |  | | |
| *lnalpha_constant* | 0.286 | | | * | | 0.354 | | ** | | 0.329 | | ** | | 0.317 | | ** | | 0.369 | | ** | | |
|  | (0.156) | | |  | | (0.151) | |  | | (0.150) | |  | | (0.147) | |  | | (0.153) | |  | | |
| *Nb of observations* | 596 | | |  | | 576 | |  | | 596 | |  | | 596 | |  | | 596 | |  | | |
| *Nb of groups* | 30 | | |  | | 30 | |  | | 30 | |  | | 30 | |  | | 30 | |  | | |
| *R^2^_pseudo* | 0.1106 | | |  | | 0.0981 | |  | | 0.1075 | |  | | 0.1084 | |  | | 0.1038 | |  | | |
| *Loglikelihood* | -3040 | | |  | | -3037 | |  | | -3051 | |  | | -3048 | |  | | -3064 | |  | | |

Note 1: ***, ** and * show significance at the 1%, 5% and 10% levels, respectively, and standard errors are presented in parentheses.
